# Kinetic Rationalization of Nonlinear Effects in Asymmetric Catalytic Cascade Reactions under Curtin–Hammett Conditions

**DOI:** 10.1021/acscatal.2c00783

**Published:** 2022-04-29

**Authors:** Camran Ali, Donna G. Blackmond, Jordi Burés

**Affiliations:** † Department of Chemistry, The University of Manchester, Manchester M13 9PL, U.K.; ‡ Scripps Research, Department of Chemistry, La Jolla, California 92037, United States

**Keywords:** nonlinear effects, reaction mechanisms, organocatalysis, asymmetric catalysis, cascade reactions, Curtin−Hammett conditions

## Abstract

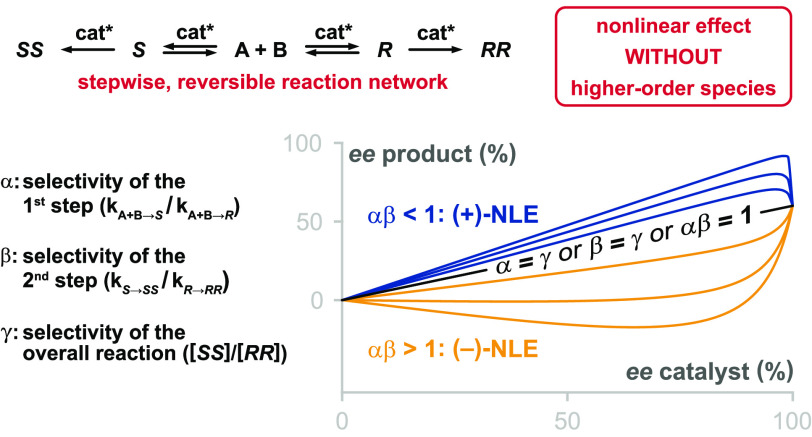

Observations of nonlinear effects of catalyst enantiopurity on product enantiomeric excess in asymmetric catalysis are often used to infer that more than one catalyst species is involved in one or more reaction steps. We demonstrate here, however, that in the case of asymmetric catalytic cascade reactions, a nonlinear effect may be observed in the absence of any higher order catalyst species or any reaction step involving two catalyst species. We illustrate this concept with an example from a recent report of an organocatalytic enantioselective [10 + 2] stepwise cyclization reaction. The disruption of pre-equilibria (Curtin–Hammett equilibrium) in reversible steps occurring prior to the final irreversible product formation step can result in an alteration of the final product *ee* from what would be expected based on a linear relationship with the enantiopure catalyst. The treatment accounts for either positive or negative nonlinear effects in systems over a wide range of conditions including “major-minor” kinetics or the more conventional “lock-and-key” kinetics. The mechanistic scenario proposed here may apply generally to other cascade reaction systems exhibiting similar kinetic features and should be considered as a viable alternative model whenever a nonlinear effect is observed in a cascade sequence of reactions.

## Introduction

**Figure 1:**
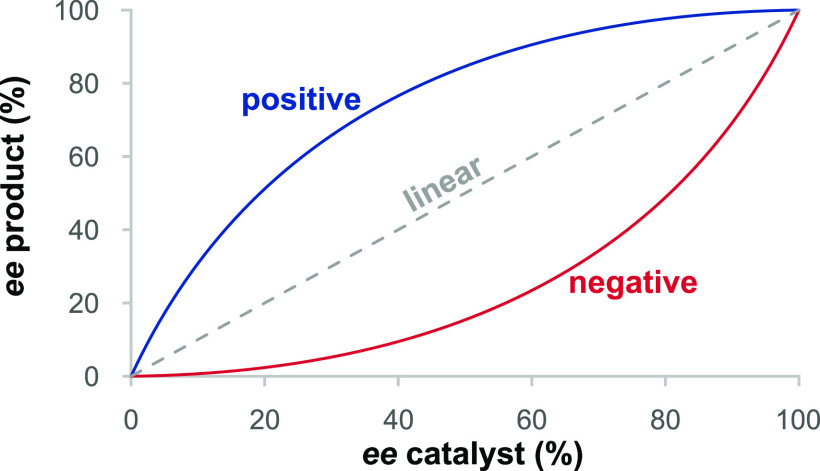
Examples of possible relationships between catalyst *ee* and reaction product *ee* in asymmetric catalytic reactions.

Probing for nonlinear effects (NLE)^1−3^ in asymmetric catalytic reactions has become a standard mechanistic tool to help understand reaction networks in which higher order catalyst species may be involved. Reactions carried out using different concentrations of two catalyst enantiomers may show either a linear relationship between the catalyst and product *ee*, suggesting that the two catalyst enantiomers act independently, or that a nonlinear relationship may exhibit either higher (positive effect) or lower (negative effect) product *ee* than that expected from the catalyst *ee* (Figure 1).

It is commonly assumed that the observation of a nonlinear effect implies that more than one catalyst molecule is involved in the enantio-determining transition state, and Kagan et al. and Blackmond’s earliest ML_*n*_ models^1,2^ treated such cases. However, observations of nonlinear effects have also been attributed to a variety of other mechanistic scenarios. For example, nonlinear effects due to catalyst monomer-dimer equilibria are not uncommon, with active monomer species and homochiral and heterochiral dimeric species that reside off-cycle. Such systems date back to one of the earliest examples, in which a striking positive nonlinear effect was observed in the dialkylzinc alkylation of aldehydes catalyzed by amino alcohols first reported by Oguni et al.^4^ and studied extensively by Noyori and coworkers.^5^ Negative nonlinear effects due to off-cycle exclusively of homochiral bis-ligated Rh and Pd catalysts have been reported in 1,4-conjugate additions^6^ and in C(sp^3^)–H functionalizations,^7^ respectively. A general protocol for determining speciation in asymmetric catalysis using both kinetics and nonlinear effects has been developed for transition-metal–chiral-ligand systems.^8^ In addition, nonlinear effects due to the phase behavior of incompletely solubilized non-enantiopure catalyst systems have mistakenly been attributed to the formation of higher order solution phase catalytic species.^9^ These cases of nonlinear effects arise from disparate chemical and physical mechanisms, but they have in common the key concept of catalyst or ligand aggregation, which allows for a distortion of the enantiomeric excess of the active fraction of the chiral catalyst compared to the total concentration of the chiral component.

Nonlinear effects have only rarely been discussed in the context of complex organocatalytic cascade reaction sequences. Over the past two decades, focus of the development of enantioselective organocatalytic cascade or domino reactions has been on synthetic strategies for increasing molecular complexity in both natural products and designed molecules.^10^ Multicomponent domino or cascade reactions have successfully been employed in organocatalytic networks to set multiple stereocenters in a consecutive sequence of reactions. A powerful and efficient tool in organic synthesis was developed, combining different activation modes to induce both high efficiency and complexity; such cascade networks have variously been termed “a new paradigm for target-oriented synthesis”^10a^ and part of “a new age of organic synthesis”.^10b^ In particular, the combination of iminium and enamine catalyses has been noted as promising sequential steps, following an early report by Enders et al. that highlighted the control of four stereocenters in a Michael/Michael/aldol condensation sequence employing diarylprolinol ether catalysts.^10c^ It has been suggested that the design of future cascade reaction networks will be based on discovering new modes of substrate activation by asymmetric organocatalysts.^10a^ While most cascade networks have involved a combination of intermolecular and intramolecular reactions, extension to multi-step, fully intermolecular sequences remains a priority of future research.^10d^ Mechanistic studies of such systems have not been extensively reported but could offer valuable information for future development.

As discussed above, probing for nonlinear effects in asymmetric catalysis can be a key mechanistic tool, and it is one that could be applied to help understand cascade reaction networks. However, we propose that due to the kinetic complexity of these systems, observation of a nonlinear effect may occur without invoking either higher order species or reactions involving two catalyst molecules. To illustrate this proposed mechanism, we apply it to the system studied in a recent literature report^11^ of a cascade sequence that invoked dual catalyst activation to rationalize an observed nonlinear effect. We demonstrate that nonlinear behavior in such a case may arise purely due to reversibility in the reaction network coupled with disruption of pre-equilibria connecting the two enantiomeric product channels. Understanding the origin of such nonlinear effects, including distinguishing between a model such as that presented here and proposals of dual catalyst species, could be a key to the design of future asymmetric catalytic cascade systems.

## Background

**Scheme 1:**
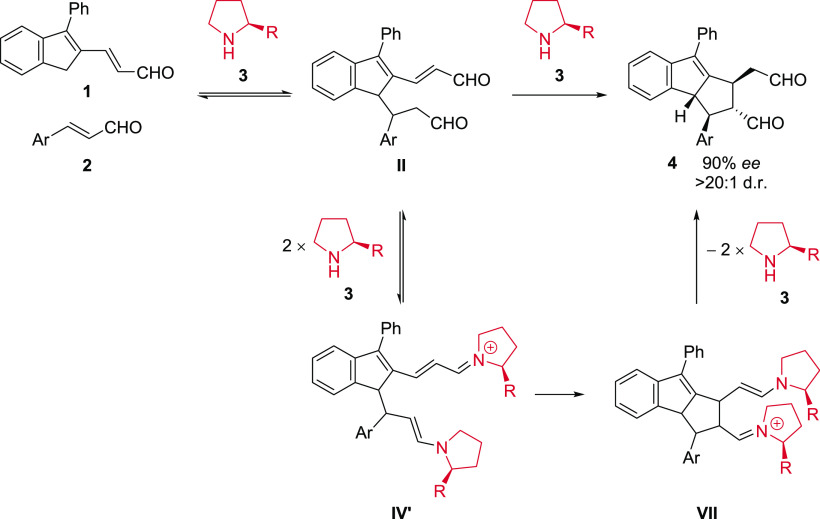
[10 + 2] Cycloaddition Reaction Catalyzed by **3** (R = diphenyl-OTMS) via Intermediate Product **II** and Dual Activation Catalytic Intermediates **IV**′ and **VII**(11)

Recently, Jørgensen and coworkers^11^ developed an organocatalytic [10 + 2] cascade cycloaddition with high formal peri-, diastereo-, and enantioselectivity (Scheme 1, compound labels from ref (11)) in which they also reported experimental and computational mechanistic studies, including observation of an unusual negative nonlinear effect. The authors interpreted this result to indicate that more than one molecule of catalyst **3** is involved in the enantio-differentiating transition state.

The authors of ref (11) proposed that intermediate product **II** forms from condensation of substrates **1** and **2** with separate molecules of catalyst **3**. Intermediate **II** then reacts further with two molecules of catalyst **3** to form **IV**′, which in turn cyclizes to produce **VII** in a “dual activation pathway”. The reaction system shown in Scheme 1 was proposed to involve a Curtin–Hammett scenario,^12^ where all diastereomers of intermediate product **II** are reversibly formed, but only the enantiomers leading to product **4** react in the final cyclization reaction.

None of the proposed catalytic intermediates in ref (11) has been detected experimentally. While DFT calculations were employed to study catalytic intermediates and transition states involved in the proposed dual activation mechanism, these calculations were reported only for the enantiopure catalyst and thus only for homochiral dual catalyst species. No molecular-level interpretation of the sense and magnitude of the nonlinear effect was offered. The current work demonstrates that the mechanism shown in calculations in ref (11) necessarily can produce only a positive, and not a negative, nonlinear effect. The alternative model presented here rationalizes both the sense and the magnitude of the observed nonlinear effect without invoking dual catalyst species such as **IV**′ and **VII**. Further, we present a general treatment showing how the model can account for either positive or negative nonlinear effects, or for linear behavior, simply due to the relative magnitude of the rate constants in the parallel-sequential cascade reaction network.

## Results and Discussion

**Scheme 2:**
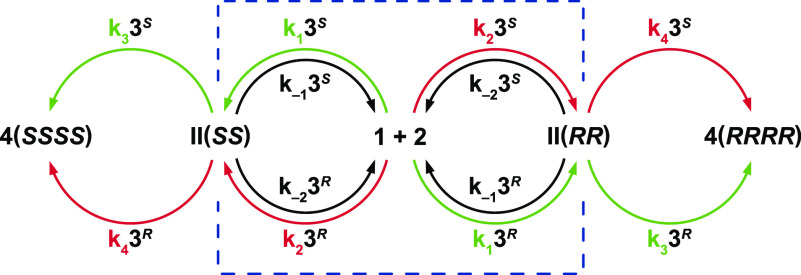
Proposed Stepwise Reaction Network for the Reaction of Scheme 1 in the Absence of a Dual Catalyst Step^15^

Scheme 2 proposes an alternate mechanism for the reaction system presented in Scheme 1 and ref (11), with the key difference being the absence of any reaction occurring between two catalyst molecules or any species containing two catalyst molecules. In the studies of ref (11), intermediate **II** was isolated and separated into two diastereomers at a ratio of 1:1.2. Separate reactions of the two isolated diastereomers of **II** gave a single diastereomer and the same *ee* for product **4** as did the reaction from **1** and **2**. Reversion of **II** back to the starting reactants **1** and **2** was also observed in the reactions initiated from **II**. These experimental observations suggest that the diastereomers of **II** react onward to form **4(*SSSS*)** and **4(*RRRR*)** solely through the reaction of catalyst **3** with enantiomers **II(*SS*)** and **II(*RR*)**, respectively. Based on these observations, and for simplicity in visualizing the network, Scheme 2 treats a system that proceeds with diastereoselective formation of only the enantiomers **II(*SS*)** and **II(*RR*)** followed by their diastereoselective conversion to products **4(*SSSS*)** and **4(*RRRR*)**. The reaction proceeds with enantiopure catalyst **3** through either the top half or bottom half of Scheme 2, or with both enantiomers of catalyst **3** in the full scheme. The full system, where all diastereomeric species are allowed to form, gives results consistent with those presented here and is treated in the Supporting Information.^13,14^

All reactions in the network within the blue dashed lines in Scheme 2 are reversible, culminating in the formation of **II(*SS*)** and **II(*RR*)**. Compound **II** is an intermediate product of the reactions and does not contain catalyst **3**. The final cyclized product **4** is formed irreversibly in a reaction catalyzed by **3** and in which catalyst **3** is regenerated. In Scheme 2, we place catalyst **3** combined with the rate constant over the reaction arrow in each step to emphasize the role of catalyst concentration in effectively increasing the rate constant for any step in which it participates. For the purposes of our simulations, we designate **4(*SSSS*)** and **3^*S*^** as the major enantiomers of the product and catalyst, respectively, which defines a major pathway shown in green and a minor pathway shown in red in Scheme 2. Although the two pathways may exhibit different catalytic kinetics under out-of-equilibrium conditions, the equilibrium condition describing the major pathway within the blue envelope is identical to that of the minor pathway. Microscopic reversibility dictates that only three of the four rate constants within the blue envelope are independent.^16^ Note that the rate constants *k*_1_ and *k*_3_ shown in green for the pathway forming **II(*SS*)** and **4(*SSSS*)** using catalyst **3^*S*^** are necessarily mirrored in the pathway forming **II(*RR*)** and **4(*RRRR*)** using catalyst **3^*R*^**. The same is true for the rate constants *k*_2_ and *k*_4_ shown in red in the pathways to product **4**.

We define the parameter α (eq 1a) as the selectivity ratio for the major vs minor pathways to form **II** from **1** and **2** and the parameter β (eq 1b) as the selectivity ratio for the major vs minor pathways to form product **4** from **II** (eq 1b). The parameter γ (eq 1c) represents the ratio of the major product **4** to the minor product **4** for the case of an enantiopure catalyst and hence serves as an overall selectivity factor for the full network. The *ee* of product **4** observed experimentally using enantiopure **3** is given by *ee*_**4**_^*ep*^ and that expected under Curtin–Hammett equilibrium conditions is given by *ee*_**4**_^*ep* (C–H)^.
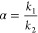
1a
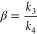
1b
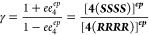
1c

We propose here that the nonlinear effect observed in the study in ref (11) may result directly from the complex network of reversible reactions shown in Scheme 2 in the absence of any reactions or intermediates involving two catalyst species. A number of different scenarios can lead to this behavior. Here, we treat two cases of the mechanism shown in Scheme 2 in simulations based on the set of rate constants shown in Tables 1 and 2, chosen to mimic the global reaction rates reported in ref (11). These findings are not limited to the reaction system of ref (11) but may be applicable to any asymmetric catalytic cascade system displaying similar kinetic features.

**Table 1:** Constants Employed in Simulations for Case 1 of the Reaction Network Shown in Scheme 2(13,17)

rate constant	value (units)	parameter	value (units)
*k*_1_	3.1143 (M^–2^ min^–1^)	α	0.58
*k*_–1_	3.9805 (M^–1^ min^–1^)	β	56.7
*k*_2_	5.3797 (M^–2^ min^–1^)	*ee***_4_***^ep^*	90 (*S*, %)
*k*_–2_	6.8759 (M^–1^ min^–1^)	*ee***_4_**^*ep* (C–H)^	97 (*S*, %)
*k*_3_	8.1453 (M^–1^ min^–1^)	γ	19.0
*k*_4_	0.1437 (M^–1^ min^–1^)		

**Table 2:** Constants Employed in Simulations for Case 2 of the Reaction Network Shown in Scheme 2(13,17)

rate constant	value (units)	parameter	value (units)
*k*_1_	9.0003 (M^–2^ min^–1^)	α	56.7
*k_–1_*	9.9037 (M^–1^ min^–1^)	β	0.58
*k*_2_	0.1587 (M^–2^ min^–1^)	*ee***_4_***^ep^*	90 (*S*, %)
*k*_–2_	0.1747 (M^–1^ min^–1^)	*ee***_4_**^*ep* (C–H)^	27 (*R*, %)
*k*_3_	4.8398 (M^–1^ min^–1^)	γ	19.0
*k*_4_	8.3603 (M^–1^ min^–1^)		

Reports of asymmetric catalytic reactions involving dual catalyst activation, as proposed in ref (11) and Scheme 1, are rare. Most prominently, Jacobsen’s epoxide ring opening is a well-documented example of a bimolecular asymmetric catalyst step.^18^ More recently, photoredox catalysis has been demonstrated to operate through interactions between a photoredox catalytic cycle and a chemical catalytic cycle, but typically, it is only the chemical cycle that includes an asymmetric catalyst.^19^ Hong and coworkers have proposed dual activation by two organocatalyst molecules in several cycloaddition reactions in total synthesis applications, without isolating intermediates, carrying out nonlinear effects studies, or providing kinetic, spectroscopic, or computational support.^20^ Kagan et al. and Puchot and Agami initially proposed a two-proline mechanism in the Hajos–Parrish–Eder–Sauer–Wiechert intramolecular aldol reaction due to the observation of a negative nonlinear effect,^1a,21^ but that reaction was later conclusively demonstrated to exhibit linear behavior, and both experimental and computational data now support a mechanism involving a single organocatalyst molecule.^22^ The nonlinear effect originally observed was then shown to arise from phase behavior considerations, with formation of a solid-phase “kinetic conglomerate” due to the low solubility of proline in DMF.^9b^

In the organocatalytic reaction of Scheme 1, solubility considerations are not likely to influence the product enantiomeric excess. However, kinetic considerations in the reversible formation of catalyst-free intermediate product **II**, followed by its re-engagement with the catalyst to undergo irreversible cyclization, make this system less straightforward to analyze than common asymmetric catalytic cycles. The reversibility within the network of reactions from substrates **1** and **2** to intermediate product **II** suggests that the onward reaction of **II** with catalyst **3** proceeds essentially as a complex dynamic kinetic resolution exclusively of the enantiomers of **II** that go on to form product **4**. Irreversible formation of **4** ultimately funnels all the reversibly formed diastereomers of **II** toward the reactive enantiomeric **II** species, further complicating the analysis beyond that of a simple dynamic kinetic resolution that typically commences with fixed (usually equal) initial concentrations of interconverting enantiomeric substrates.

**Figure 2:**
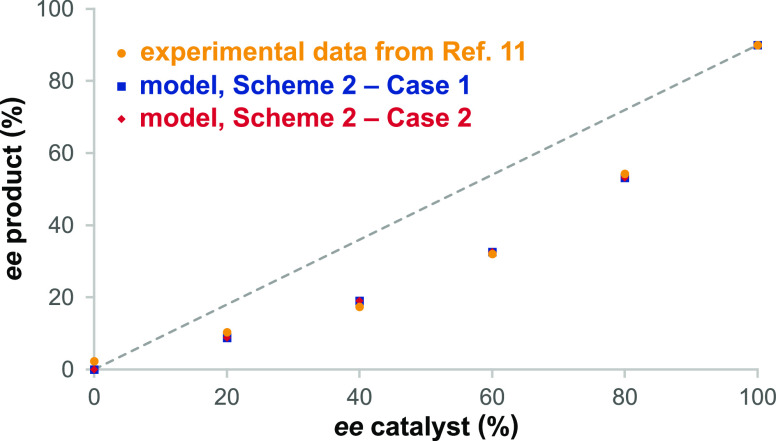
Enantiomeric excess of product **4** as a function of the enantiopurity of catalyst **3**. Experimental values from ref (11) (orange circles); simulations based on the reaction network of Scheme 2 employing the rate constants from Table 1 (Case 1, blue squares) and Table 2 (Case 2, red diamonds); the linear relationship is given by the dashed line. Conditions: [**1**]_0_ = 0.125 M, [**2**]_0_ = 0.25 M, and [**3**]_total_ = 0.025 M.

Simulations^13,17^ show that the reaction network of Scheme 2 employing the sets of constants given in Tables 1 and 2 each reproduce the trends for the reaction of Scheme 1 as reported in ref (11). First, for case of the enantiopure catalyst **3^*S*^**, the simulations give the enantiomeric excess of product **4(*SSSS*)**, *ee*_**4**_^*ep*^, at ca. 90% *ee* as was found experimentally. Second, applying the model to the reaction initiated from mixtures of the diastereomers of **II**, formation of the starting materials **1** and **2** is observed, confirming the observed reversibility in the network. Third, as shown in Figure 2, when simulations of the reaction network of Scheme 2 are carried out for Cases 1 and 2 using varying concentrations of the two enantiomers of catalyst **3**, a negative nonlinear effect identical to that reported in ref (11) is observed, in this case with no reaction step nor any intermediate species involving two catalyst molecules.

**Figure 3:**
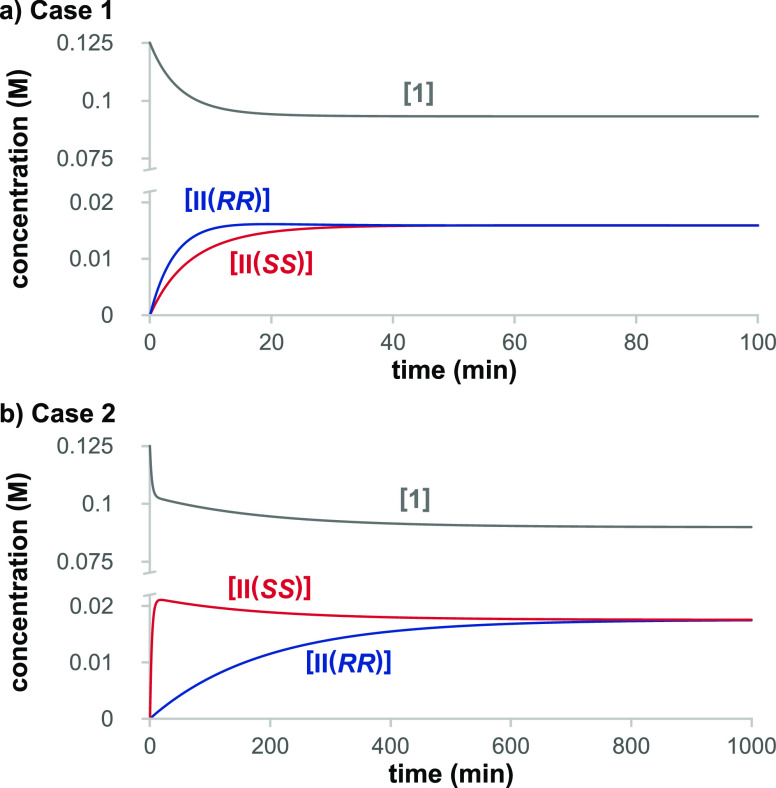
Simulation of the reversible formation of **II(*RR*)** and **II(*SS*)** using catalyst **3^*R*^** in the network in Scheme 2 within the blue envelope in the case where **II** cannot react further to form **4** (*k*_3_ and *k*_4_ set equal to 0); (a) Case 1 from Table 1; (b) Case 2 from Table 2. Conditions: [**1**]_0_ = 0.125 M, [**2**]_0_ = 0.25 M, and [**3^*S*^**] = 0.025 M.

Mechanistic insights into the origin of a nonlinear effect in the absence of higher order species may be found by studying each of the model scenarios Cases 1 and 2 in detail. The reaction network shown in Scheme 1 was proposed in ref (11) to operate under Curtin–Hammett equilibrium conditions. It is important first to understand features of the reversible reaction network inside the blue envelope of Scheme 2 by establishing the theoretical equilibrium condition between the starting materials and intermediate products **II(*RR*)** and **II(*SS*)**. A key consideration is that under conditions where all the reversible reactions are in equilibrium, all species within the blue envelope will be formed in their thermodynamically dictated ratios ([**II(*SS*)**] = [**II(*RR*)**]), regardless of whether enantiopure or mixed enantiomer catalysts are employed. The magnitude of rate constants *k*_3_ and *k*_4_ can cause perturbations of the pre-equilibria and can alter the observed concentrations of species within the blue envelope, but they cannot alter the theoretical equilibrium condition, which is dictated by the values of *k*_1_, *k*_–1_, *k*_2_, and *k*_–2_. The theoretical equilibrium condition for **II** in the network of Scheme 2 is revealed in simulations by temporarily and artificially removing the irreversible steps from **II** to product **4**, including only the reversible reactions within the blue envelope (setting *k*_3_ = *k*_4_ = 0) in the simulations. Figure 3 shows that at equilibrium, the concentration of [**1**] is significant in both Cases 1 and 2, corroborating the experimental observation that reactions to form **II** are reversible.^11^Figure 3 also shows that the two enantiomers of **II** show different trends in their approach to equilibrium in the two cases. In Case 1, enantiomer **II(*RR*)**, leading to the minor product **4(*RRRR*)**, forms more rapidly than does **II(*SS*)**. In Case 2, enantiomer **II(*SS*)** leading to the major product forms more rapidly, initially overshooting its equilibrium concentration, while **II(*RR*)** rises much more slowly. Case 2 requires a significantly longer time to approach equilibrium than does Case 1, only attaining equilibrium near the end of the reaction time reported in ref (11).

The implications of this approach to equilibrium become important when the reaction steps to form product **4** (rate constants *k*_3_ and *k*_4_) are included in the simulations. In the scenario proposed in ref (11), where enantiomers **II(*RR*)** and **II(*SS*)** proceed on to product **4** under the equilibrium Curtin–Hammett conditions, enantioselectivity for product **4**, *ee*_**4**_^*ep* (C–H)^, must arise under kinetic control due to differences between the irreversible rate constants in the final cyclization step, *k*_3_ and *k*_4_ (where *ee*_**4**_^*ep* (C–H)^ = (*k*_3_ – *k*_4_)/(*k*_3_ + *k*_4_)). From Tables 1 and 2, we calculate that under equilibrium conditions for the formation of **II**, the *ee* of product **4** using an enantiopure catalyst, *ee*_**4**_^*ep* (C–H)^, would be 97% *ee* toward **4(*SSSS*)** in Case 1 and 27% *ee* toward the opposite product **4(*RRRR*)** in Case 2. The fact that these values differ from the 90% *ee* toward **4(*SSSS*)** found both experimentally and in the full reaction simulations confirms that in both Cases 1 and 2, the system in Scheme 2 proceeds with some of the reversible reactions perturbed from equilibrium status. Interestingly, in Case 1, the experimental *ee*_**4**_ value is lower, while in Case 2, the experimental *ee*_**4**_ is significantly higher, and opposite in sense, than that predicted for the reaction network under Curtin–Hammett equilibrium control. In fact, as described below, this perturbation from equilibrium resulting in deviation from the enantioselectivity predicted from the irreversible product forming step in the enantiopure case is the basis for the nonlinear effect observed in Figure 2.

**Figure 4:**
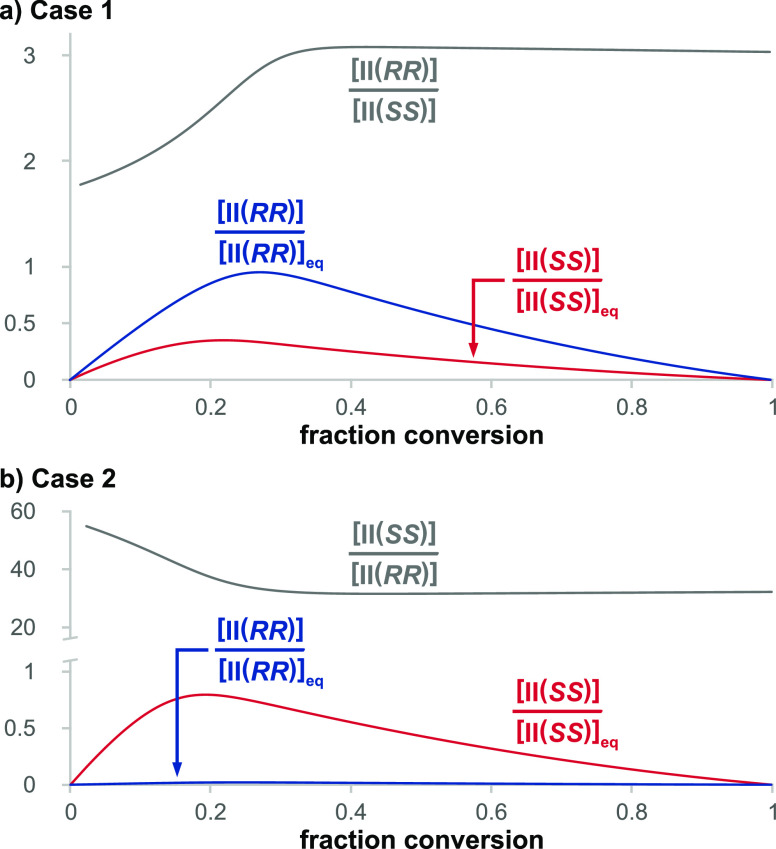
Simulation of the full reaction network in Scheme 2 to form product **4** for enantiopure catalyst **3^*S*^** in (a) Case 1 and (b) Case 2. Fraction of the equilibrium concentration of **II(*RR*)** (blue) and **II(*SS*)** (red) attained by the system as a function of conversion to product **4** and the ratio of the major to minor species of **II** (gray). Conditions: [**1**]_0_ = 0.125 M, [**2**]_0_ = 0.25 M, and [**3^*S*^**] = 0.025 M.

This perturbation from equilibrium persists throughout the reaction, quantified as shown in Figure 4 for the full reaction network of Scheme 2 with enantiopure catalyst **3^*S*^** in Case 1 (Figure 4a) and in Case 2 (Figure 4b). In Case 1, the concentration of **II(*RR*)**, leading to the minor product **4(*RRRR*)**, dominates, rising to a maximum at over 80% of its equilibrium concentration early in the reaction before decaying at conversions higher than 20% as product **4** is formed. By contrast, the concentration of **II(*SS*)** leading to the major product **4(*SSSS*)** rises only to ca. 30% of its equilibrium value under these conditions. The model shows further that throughout the reaction, the relative concentration of the enantiomer of **II** leading to the minor product of **4** compared to the major product remains a factor of ca. 3 higher than that predicted for the case where the reversible reactions are under equilibrium. The departure from equilibrium is even starker in Case 2, where **II(*SS*)** leading to the major product dominates, and **II(*RR*)** attains less than 3% of its equilibrium concentration. In contrast to Case 1, the ratio of the enantiomers of **II** exceeds 30:1 in favor of the major product channel in Case 2.

In both cases shown in Figure 4, this reaction network effectively operates as a “distorted” dynamic kinetic resolution where the interconverting enantiomers of **II** are not present as a racemic mixture but instead maintain a non-zero *ee*. As the reaction progresses in Case 1, **II***ee*_**II**_ rises to ca. 50% toward **II(*RR*)**, and for Case 2, the system stabilizes at ca. 94% *ee*_**II**_ toward **II(*SS*)**. It is the reversibility of the reactions within the blue envelope together with the perturbation from equilibrium of these reactions that allows the system to sustain unequal concentrations of the enantiomers of **II**.

Experimental and computational studies of kinetic resolutions employing nonenantiopure catalysts have highlighted the potential for mechanistic insight into these systems.^23^ In a number of cases, nonlinear effects in kinetic resolutions have been documented in mechanisms that do not involve dual catalyst steps. Ismagilov found that inaccurate selectivity factors may be obtained in kinetic resolutions carried out with either nonracemic substrates and/or nonenantiopure catalysts and showed how to correct these factors.^23a^ Lloyd-Jones and coworkers exploited similar concepts in kinetic resolutions using racemic catalysts and nonenantiopure substrates under pseudo-zero order conditions in substrate concentration as a method for screening catalysts for selectivity without the need to separate the catalyst enantiomers.^23e^ Blackmond demonstrated that selectivity factors in kinetic resolution can become conversion-dependent due to “kinetic partitioning” of catalysts within complex reaction networks.^23d^ Kalek and Fu treated the case of nonlinear effects in irreversible enantioconvergent kinetic resolutions, revealing that the magnitude of an intrinsically negative nonlinear effect correlated with selectivity factor and conversion, without the involvement of higher order species or dual activation pathways.^23f^ The reaction network under consideration in the present work differs from these cases in that it describes a cascade sequence of reactions in which an intermediate product is reversibly formed and then re-engages with the catalyst for a further irreversible reaction step. In such a case, the potential exists for sequential selection steps that bear a resemblance to a Horeau amplification^24^ (or depletion) mechanism.

The mechanism in Scheme 2 for Case 1, where the dominant species **II(*RR*)** leads to the minor product, bears a resemblance to the “major-minor” concept developed by Landis and Halpern^25^ to rationalize changes in enantioselectivity with changes in hydrogen pressure in the Rh phosphine-catalyzed asymmetric hydrogenation of enamides. Under the Curtin–Hammett (low pressure) limit, substrate binding remains in pre-equilibrium in both enantiomeric product channels. At higher pressures, a perturbation in the substrate binding pre-equilibria may occur to a greater extent on one product pathway compared to the other. Under “major-minor” conditions, the intermediate concentration on the major product channel decreased relative to that of the minor product channel, resulting in a decrease in product *ee* with increasing pressure. At the time, this finding was an unusual observation because it is contradictory to conventional “lock-and-key” kinetics. Figure 4a shows that the “major-minor” concept introduced in asymmetric hydrogenation applies in Case 1 of the reaction network of Scheme 2 under the conditions of Table 1. The greater perturbation from equilibrium on the **II(*SS*)** channel leading to the major product results in the threefold shift away from the expected equal concentrations of the enantiomers of **II** toward **II(*RR*)** on the minor product pathway. This in turn results in a comparatively smaller concentration driving force on the major product pathway, a scenario that rationalizes the observation of a product *ee*_**4**_^*ep*^ for the enantiopure catalyst that is lower than *ee*_**4**_^*ep* (C–H)^ predicted from the Curtin–Hammett equilibrium scenario based on the relative magnitudes of *k*_3_ and *k*_4_.

The example of Case 2 demonstrates that observation of a negative nonlinear effect in the reaction network of Scheme 2 is not restricted to a “major-minor” scenario but may also be observed under more conventional “lock-and-key” kinetics, where the major enantiomer leads to the major product. Figure 4 (bottom) shows that in this case, the major species of **II** is the **II(*SS*)** intermediate leading to the major product **4(*SSSS*)**. The much larger perturbation from equilibrium for the minor intermediate **II(*RR*)** in Case 2 means that it never attains a sufficiently high rate of product formation because its concentration is continually shifted to the major species **II(*SS*)** in the reversible network within the blue envelope in Scheme 2. In this case, the perturbation from equilibrium conditions results in a reversal in sense and a strong enhancement in the magnitude of the *ee* for the enantiopure catalyst compared to that expected from rate constants *k*_3_ and *k*_4_ under Curtin–Hammett equilibrium.

The perturbation of equilibria in the reactions within the blue envelope in Scheme 2 also occurs in reactions employing nonenantiopure catalysts. In this case, molecules of **1** and **2** navigate reversibly back and forth not only along the major and minor pathways of one hand of the catalyst (either the upper half or the lower half of Scheme 2), but they also cross over between enantiomeric catalyst channels. It is this capacity for crossover from one catalyst to the other, coupled with perturbation from Curtin–Hammett conditions, that allows for nonlinear effects to be observed in this network.

**Figure 5:**
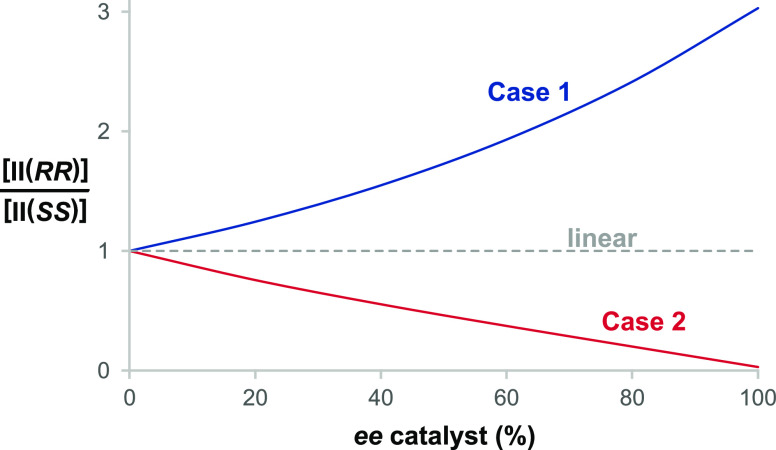
Ratio of **[II(*SS*)]/[II(*RR*)]** as a function of catalyst *ee* for Cases 1 and 2 of the model shown in Scheme 2 and eq 2. The value of unity gives linear behavior.

In both Cases 1 and 2, the reaction network attains a constant, non-unity ratio of **II(*RR*)** to **II(*SS*)** over the course of the reaction. The enantiomeric excess of product **4**, *ee*_**4**_, depends on this ratio, the rate constants *k*_3_ and *k*_4_, and the concentrations of each catalyst enantiomer, as shown in eq 2 for systems under steady-state catalysis.^13^ In a simple dynamic kinetic resolution, the ratio of **II(*RR*)**/**II(*SS*)** equals unity and remains unchanged when catalyst enantiomeric excess is altered, giving linear behavior. Under the conditions of Cases 1 and 2, where the equilibria within the blue envelope of Scheme 2 are perturbed, the ratio of [**II(*RR*)**]/[**II(*SS*)**] does not equal unity and does not remain constant as catalyst enantiomeric excess changes (Figure 5). The non-unity ratio of **[II(*RR*)]/[II(*SS*)]** gives rise to the observed nonlinear effect on the enantiomeric excess of product **4**.
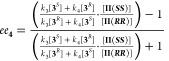
2

Note that the factors α and β in Tables 1 and 2, which represent the selectivity ratios for the sequential steps in the mechanism of Scheme 2, are interchanged in Cases 1 and 2, while the product αβ remains the same. Under these special conditions, the observed nonlinear effect is identical in sense and magnitude, with one case exhibiting major-minor kinetics and the other giving lock-and-key. Further study of the parameters α, β, and γ helps to shed light on the nonlinear effect as a general phenomenon beyond the specific conditions of Cases 1 and 2, as shown in Table 3. As mentioned previously, α, β, and γ represent selectivity factors for the first step, the second step, and the overall network, respectively. The relative magnitudes of these three parameters determine the kinetic scenario (major-minor vs lock and key), while the parameter αβ, representing the product of the two sequential steps, dictates the sense of the nonlinear effect.

**Table 3:** Outcome of Reactions Perturbed from Equilibrium in the Network of Scheme 2

α, β, and γ relationship	kinetic scenario	sense of the nonlinear effect	
α > γ > β	lock and key (e.g., Case 2)	αβ > 1	(−) NLE
		αβ = 1	linear
		αβ < 1	(+) NLE
α = γ ≠ β	irreversible	linear	
β = γ ≠ α	quasi-equilibrium	linear	
α = β = γ	equal selectivity in each step	linear	
α < γ < β	major-minor (e.g., Case 1)	αβ > 1	(−) NLE
		αβ = 1	linear
		αβ < 1	(+) NLE

Linear behavior is expected in several limiting cases. When the product αβ = 1, the distortion in selectivity arising in the first selection step is balanced by an opposite effect in the second selection step, resulting in linear behavior for the overall network. If each step has identical selectivity (α = β = γ), it results in linear behavior. In the case where the reversible reactions within the blue envelope in Scheme 2 remain in equilibrium, the selectivity of the overall reaction network would be determined by the selectivity of the second step (γ = β) and no nonlinear effect would be observed. Linear behavior would also be observed in the case where all the reactions within the blue envelope are irreversible, and therefore selectivity in the network is dictated by the first step (α = γ). In that case, the connection between the reaction channels for the two enantiomers is cut off, and perturbation in the relative concentrations of **II(*RR*)** and **II(*SS*)** due to crossover between the channels cannot occur. Nonlinear behavior results in all other cases where αβ is either greater or less than one, demonstrating the generality of the model.

**Figure 6:**
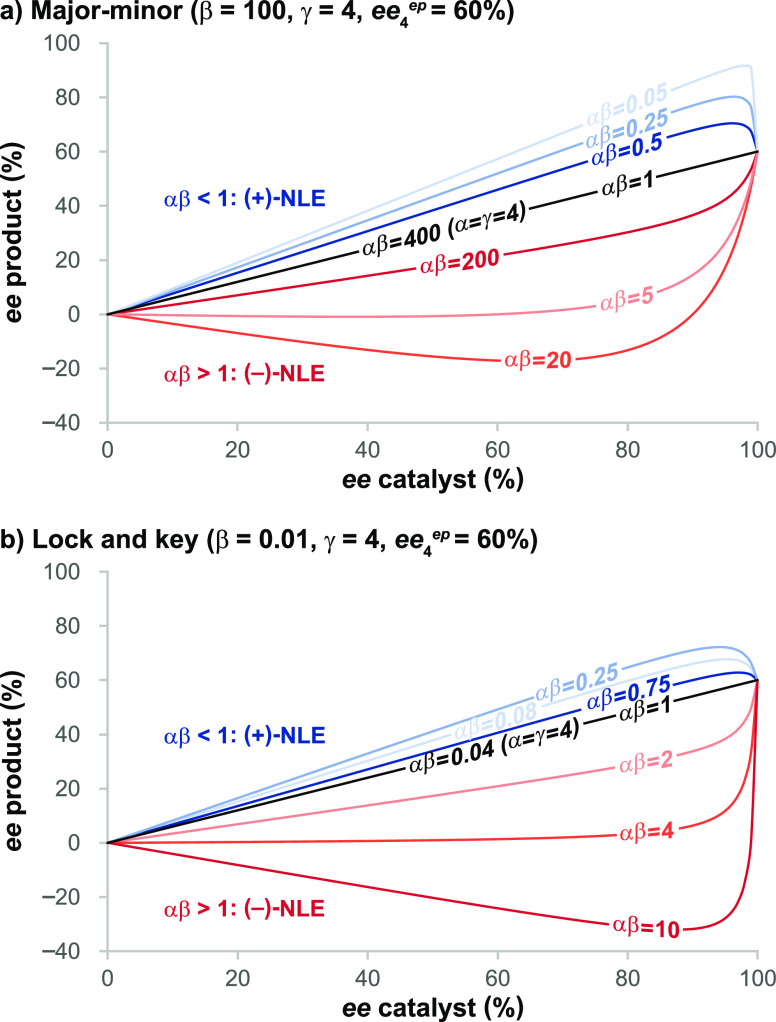
Product *ee* as a function of catalyst *ee* for a reaction in which *ee*_**4**_^*ep*^ = 60% (γ = 4) for a variety of values of α and β (eq 1 and Table 3); (a) major-minor; (b) lock-and key. Further examples are provided in the Supporting Information.^13,17^

Figure 6 illustrates the general relationships in Table 3, plotting product *ee* vs catalyst *ee* for the case of a reaction following the mechanism in Scheme 2 in which *ee*_**4**_^*ep*^ = 60%. Major-minor (Figure 6a) and lock-and-key (Figure 6b) scenarios are treated. Both positive and negative nonlinear effects may be observed, and in some cases, an *ee* value higher than that obtained with the enantiopure catalyst is observed (for αβ > 1). Such “hyper-NLE” behavior was first discussed by Kagan et al.^1b^ for ML_*n*_ systems where *n* > 2, and the effect has more recently been proposed for systems in which both monomer and dimer catalysts are active.^26^ These literature examples involve higher order catalyst species, in contrast to the current work where no higher order species or bimolecular catalyst reactions occur.

Features of the present model suggest that in cases where a nonlinear effect is observed, probing the effect of catalyst concentration on the reaction order and on product *ee* may help distinguish a mechanistic proposal involving two catalyst molecules from alternate models such as that proposed here that may provide a simpler explanation. The mechanism shown in Scheme 2 obeys first-order kinetics in the catalyst concentration for reactions with either enantiopure or nonenantiopure catalysts.^13^ By contrast, in the case where dual catalyst reactions are involved, either on or off the cycle, complex deviations from first-order dependence on the catalyst concentration are often expected.^8^

Most models for nonlinear effects^1−5^ are based on mixed enantiomer catalyst systems that form homochiral and heterochiral dual catalyst species. For example, a negative nonlinear effect in a Kagan ML_2_ model^1^ implicates formation of catalytically active heterochiral species containing one molecule of each hand of the ligand, which must react faster than the homochiral species and give racemic product **4**. Such a scenario for a heterochiral dual activation is difficult to envision in the stereochemical model presented in ref (11) for cyclization of species **IV**′. Alternatively, heterochiral species might be envisioned to form as inactive off-cycle species; however, in both Kagan ML_2_^1^ and Noyori^5^ models, this would manifest as a positive nonlinear effect. A negative nonlinear effect has been observed in systems based on purely homochiral dual or higher order catalyst species,^6,7,27^ but in that case, the species do not act as active catalyst intermediates, existing as off-cycle spectator species.

**Figure 7:**
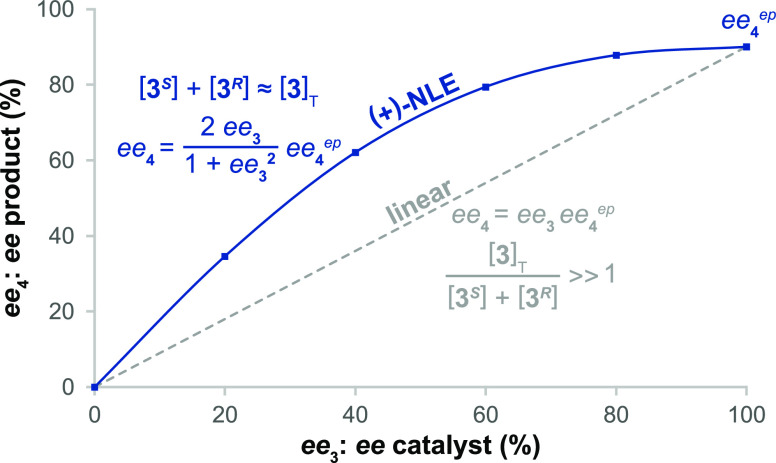
Computed reaction steps given in Figure 5 of ref (11) cannot generate negative nonlinear effects.

The reaction mechanism proposed in ref (11) invoked a complex series of steps, including three different dual catalyst species and two bimolecular catalyst reaction steps. The calculations presented in ref (11) to support the dual activation mechanism were carried out only for enantiopure catalysts, demonstrating only homochiral two-catalyst species as active species in the reaction. The computed pathway involves ring closure of homochiral species **IV**′ as the enantio- and rate-determining steps. Mathematical derivation of the rate law for this case reveals that the nonlinear effect can only be positive (eq 50, S-16).^13^ In the limiting case where the free monomeric catalyst dominates, the nonlinear effect is maximum (blue line in Figure 7), and when the catalytic species with two molecules of catalyst dominate, the enantiomeric excess of the product is linearly proportional to the enantiomeric excess of the catalyst (gray line in Figure 7).^13^

Although it was the observation of a nonlinear effect in mixed enantiomeric catalyst reactions that led to the proposed mechanism, only the case of enantiopure catalyst was studied mechanistically in ref (11). No experiments to probe the stereochemistry of catalyst intermediates in reactions featuring nonenantiopure catalysts were reported in ref (11). No two-catalyst intermediates were observed experimentally, even at significant overall catalyst concentrations. It would appear likely that if the proposed dual catalyst intermediates are feasible in the system studied in ref (11), experimental evidence for similar species would be found in other organocatalytic reactions, given that enamine and iminium ion species formed from similar substrates occur in a wide range of reported reactions catalyzed by diarylprolinol ether catalysts. Such reactions have been monitored spectroscopically and extensively characterized,^28^ but no such dual catalyst species have been reported. No model presented in the literature to date can reconcile the negative nonlinear effect reported in ref (11) with the mechanistic steps proposed in that work. By contrast, the alternate model proposed here rationalizes the nonlinear effect observed in that example without invoking dual catalyst species.

Table 3 and Figure 6 demonstrate that a variety of different scenarios derived from a network with the features shown in Scheme 2 can produce nonlinear effects without invoking higher order species or any reaction step involving two catalyst molecules. A key general point from this work is the conclusion that in complex, sequential/parallel cascade reaction networks, a nonlinear correlation between the catalyst and final product *ee* may arise from purely kinetic considerations rather than from the conventional rationalization invoking two catalyst species in one or more elementary steps. The observation of a nonlinear effect may be a general feature of cascade reactions, with the key characteristics leading to nonlinear behavior being (i) the reversibility of reactions and (ii) a perturbation of these reactions from equilibrium that occurs to a greater extent in the pathway of one catalyst enantiomer in the network compared to the other, as dictated solely by the rate constants in the network.

Cascade reactions in asymmetric catalysis have been reported in a variety of different mechanistic frameworks, including transition metal-catalyzed reactions involving photoredox catalysis^19^ and organocatalytic addition/cyclizations.^10,11,20^ While few of the reported cases have searched for nonlinear effects, it is likely that many of those cases could exhibit kinetic features similar to the system described here. In such cases, employment of nonenantiopure catalysts may provide mechanistic insights and may support proposals other than dual catalyst activation, as in the example described here.

## Conclusions

Models for nonlinear effects in asymmetric catalysis often propose that two chiral catalyst molecules are involved in the reaction’s transition state. A recently published organocatalytic cascade reaction system in which a negative nonlinear effect was observed proposed such a dual-catalyst activation pathway.^11^ By contrast, the present work develops a model for rationalizing the observed nonlinear effects that involves neither the formation of higher order catalyst species nor a reaction involving two catalyst species in the same step. The model is explored through reaction simulations showing that reversible steps prior to an irreversible product forming step provide a conduit connecting the two enantiomeric product pathways. Under conditions where the equilibria of the reversible reaction steps are disrupted, an alteration of the final product *ee* may be observed compared to what would be expected if the reversible reactions remained under Curtin–Hammett equilibrium conditions. The mechanism proposed here may be general for any system exhibiting these kinetic features and should be considered as a potential alternative model whenever a nonlinear effect is observed in a cascade sequence of reactions.

## Supporting Information Available

Details of the kinetic modeling including COPASI scripts and mathematic equations for the model in Scheme 2 (PDF)
Complete model including all diastereomeric intermediate species and models for several other specific limiting conditions (ZIP)
