# Supplementary material for: Kinetic Rationalization of Nonlinear Effects in Asymmetric Catalytic Cascade Reactions under Curtin–Hammett Conditions
Source: ACS Catal. 2022 Apr 29;12(10):5776–85. doi: 10.1021/acscatal.2c00783 (PMC9127809; doi:10.1021/acscatal.2c00783)
Supplement: Supplementary file 2 — cs2c00783_si_002.pdf [file cs2c00783_si_002.pdf]

Supporting Information

Kinetic Rationalization of Nonlinear Effects in Asymmetric Catalytic Cascade Reactions Under Curtin-Hammett Conditions

Camran Ali,[1] Donna G. Blackmond,[2]* and Jordi Burés[1]*

[1]Department of Chemistry, The University of Manchester, Oxford Road, Manchester M13 9PL, UK

[2]Scripps Research, Department of Chemistry, La Jolla, CA 92037, USA

*blackmond@scripps.edu; jordi.bures@manchester.ac.uk

Table of Contents

1. General remarks S-1

2. Kinetic analysis of the nonlinear effects of the reaction network in Scheme 2 S-1

3. Procedure used to obtain rate constants for the kinetic modelling of Scheme 2 S-7

4. Kinetic modelling of Scheme 2 S-8

5. Kinetic modelling of the extended reaction with unproductive diastereomeric intermediates S-10

6. Kinetic modelling of Scheme 2 with a completely enantiospecific catalyst in the final step S-11

7. Kinetic modelling of Scheme 2 with an uncatalyzed final step S-11

8. Measurement of the order in catalyst for Scheme 2 S-12

9. Procedure for the modelling of Figure 5 S-13

10. Kinetic analysis of the mechanism shown in Ref. 2 (SI) S-14

11. References S-17

1. General remarks

All kinetic modelling was conducted using Complex Pathway Simulator (COPASI) software.[1] The values used in each kinetic model are provided, as well as the corresponding COPASI files. The data of the nonlinear effects used in the simulations have been extracted from the experimental data reported by Jørgensen in ACS catalysis 2020 for the reaction in **Scheme S1**.[2] The reaction involves a catalyzed reaction between **1** and **2**, producing intermediate **II**, which cyclises in a catalyzed reaction to from the final product **4**. All the conditions used for the COPASI modelling were extracted from page S64 in the supporting information of Ref. 2 (SI) unless stated otherwise. Within our COPASI models, we refer to **1**, **2**, and **3** as A, B, and cat respectively.

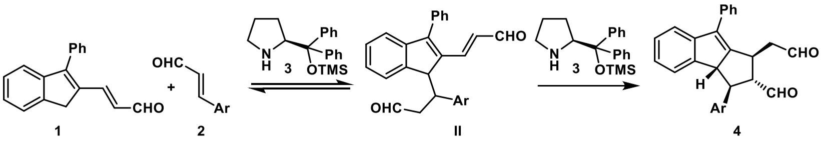

Scheme S1. Organocatalytic cycloaddition of **1** and **2** to form **4** via intermediate **II**.[2]

2. Kinetic analysis of the nonlinear effects of the reaction network in Scheme 2

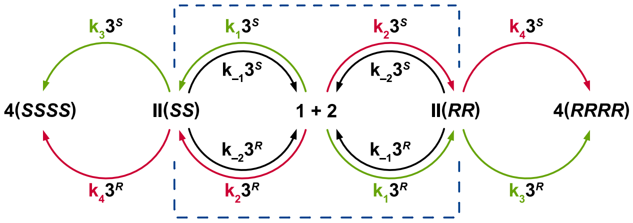

Scheme S2. Reaction scheme used for the mathematical analysis of nonlinear effects observed in cascade reactions.

We derived an equation for ee_4 by applying the steady-state approximation to the reaction intermediates **II(*SS*)** and **II(*RR*)**. We will use the following equations and definitions:

Enantiomeric ratio of product **4**:

$$er_4 = \frac{[4(SSSS)]}{[4(RRRR)]} \quad \textbf{(1)}$$

Rate of formation of **4(*SSSS*)**:

$$r_{4(SSSS)} = (k_3[3^S]+k_4[3^R])[\mathrm{II}(SS)] \quad \textbf{(2)}$$

Rate of formation of **4(*RRRR*)**:

$$r_{4(RRRR)} = (k_3[3^R]+k_4[3^S])[\mathrm{II}(RR)] \quad \textbf{(3)}$$

Steady-state approximation for **II(*SS*)**:

$$(k_1[3^S] + k_2[3^R])[1][2] - (k_{-1}[3^S] + k_{-2}[3^R] + k_3[3^S]+k_4[3^R])[\mathrm{II}(SS)] \approx 0 \quad \textbf{(4)}$$

Steady-state approximation for **II(*RR*)**:

$$(k_1[3^R] + k_2[3^S])[1][2] - (k_{-1}[3^R] + k_{-2}[3^S] + k_3[3^R]+k_4[3^S])[\mathrm{II}(RR)] \approx 0 \quad \textbf{(5)}$$

From the equilibrium of **1** and **2** with **II(*SS*)**:

$$\frac{[\mathrm{II}(SS)]_{eq}[3^S]_{eq}}{[1]_{eq}[2]_{eq}[3^S]_{eq}} = \frac{k_1}{k_{-1}} = \frac{k_2}{k_{-2}}, \text{and therefore: } k_{-2} = \frac{k_2 k_{-1}}{k_1} \quad \textbf{(6)}$$

Enantioselectivity of the first step:

$$\alpha = \frac{k_1}{k_2} \quad \textbf{(7)}$$

Selectivity factor of the second step (analogous to the selectivity factor of kinetic resolutions):

$$\beta = \frac{k_3}{k_4} \quad \textbf{(8)}$$

Enantiomeric ratio and enantiomeric excess of catalyst **3**:

$$\text{As } er_3 = \frac{[3^S]}{[3^R]} \text{ and } ee_3 = \frac{[3^S]-[3^R]}{[3^S]+[3^R]}, \text{then } \frac{[3^S]}{[3^R]} = \frac{1+ee_3}{1-ee_3} \quad \textbf{(9)}$$

Enantiomeric excess of product **4**:

$$ee_{\mathbf{4}} = \frac{[4(SSSS)]-[4(RRRR)]}{[4(SSSS)]+[4(RRRR)]} = \frac{er_{\mathbf{4}}-1}{er_{\mathbf{4}}+1} \quad \textbf{(10)}$$

From equations **(1)**, **(2)** and **(3)**:

$$er_{\mathbf{4}} = \frac{(k_3[3^S]+k_4[3^R])[\mathrm{II}(SS)]}{(k_3[3^R]+k_4[3^S])[\mathrm{II}(RR)]} \quad \textbf{(11)}$$

From equations **(11)**, **(4)** and **(5)**:

$$er_{\mathbf{4}} = \frac{(k_3[3^S]+k_4[3^R])\dfrac{(k_1[3^S]+k_2[3^R])[1][2]}{k_{-1}[3^S]+k_{-2}[3^R]+k_3[3^S]+k_4[3^R]}}{(k_3[3^R]+k_4[3^S])\dfrac{(k_1[3^R]+k_2[3^S])[1][2]}{k_{-1}[3^R]+k_{-2}[3^S]+k_3[3^R]+k_4[3^S]}}$$

$$er_{\mathbf{4}} = \frac{(k_3[3^S]+k_4[3^R])(k_1[3^S]+k_2[3^R])([3^S](k_{-2}+k_4)+[3^R](k_{-1}+k_3))}{(k_3[3^R]+k_4[3^S])(k_1[3^R]+k_2[3^S])([3^R](k_{-2}+k_4)+[3^S](k_{-1}+k_3))} \quad \textbf{(12)}$$

From equations **(12)** and **(6)**:

$$er_{\mathbf{4}} = \frac{(k_3[3^S]+k_4[3^R])(k_1[3^S]+k_2[3^R])\left([3^S]\left(\frac{k_2k_{-1}}{k_1}+k_4\right)+[3^R](k_{-1}+k_3)\right)}{(k_3[3^R]+k_4[3^S])(k_1[3^R]+k_2[3^S])\left([3^R]\left(\frac{k_2k_{-1}}{k_1}+k_4\right)+[3^S](k_{-1}+k_3)\right)}$$

$$er_{\mathbf{4}} = \frac{(k_3[3^S]+k_4[3^R])(k_1[3^S]+k_2[3^R])\left([3^S]\frac{k_2k_{-1}+k_1k_4}{k_1(k_{-1}+k_3)}+[3^R]\right)}{(k_3[3^R]+k_4[3^S])(k_1[3^R]+k_2[3^S])\left([3^R]\frac{k_2k_{-1}+k_1k_4}{k_1(k_{-1}+k_3)}+[3^S]\right)} \quad \textbf{(13)}$$

From equation **(13)** we derive the er_4 when using enantiomerically pure catalyst $\mathbf{3^S}$ ($er_4^{ep} = \gamma$):

$$er_{\mathbf{4}}^{ep} = \gamma = \frac{k_1}{k_2}\frac{k_3}{k_4}\frac{k_2k_{-1}+k_1k_4}{k_1(k_{-1}+k_3)} \quad \textbf{(14)}$$

From equation **(14)**, and definitions **(7)** and **(8)**:

$$\gamma = \alpha\beta\frac{k_2k_{-1}+k_1k_4}{k_1(k_{-1}+k_3)}$$

$$\frac{k_2k_{-1}+k_1k_4}{k_1(k_{-1}+k_3)} = \frac{\gamma}{\alpha\beta} \quad \textbf{(15)}$$

From equations **(13)**, **(15)** and definitions **(7)** and **(8)**:

$$er_4 = \frac{(k_3[3^S]+k_4[3^R])(k_1[3^S]+\ k_2[3^R])\left([3^S]\frac{\gamma}{\alpha\beta}+[3^R]\right)}{(k_3[3^R]+k_4[3^S])(k_1[3^R]+\ k_2[3^S])\left([3^R]\frac{\gamma}{\alpha\beta}+[3^S]\right)}$$

$$er_4 = \frac{(\beta[3^S]+[3^R])(\alpha[3^S]+\ [3^R])([3^S]\gamma+\alpha\beta[3^R])}{(\beta[3^R]+[3^S])(\alpha[3^R]+\ [3^S])([3^R]\gamma+\alpha\beta[3^S])} \qquad \textbf{(16)}$$

From equations **(16)** and **(9)**:

$$er_4 = \frac{\left(\beta\frac{[3^S]}{[3^R]}+1\right)\left(\alpha\frac{[3^S]}{[3^R]}+\ 1\right)\left(\frac{[3^S]}{[3^R]}\gamma+\alpha\beta\right)}{\left(\beta+\frac{[3^S]}{[3^R]}\right)\left(\alpha+\ \frac{[3^S]}{[3^R]}\right)\left(\gamma+\alpha\beta\frac{[3^S]}{[3^R]}\right)}$$

$$er_4 = \frac{\left(\beta\frac{1+ee_3}{1-ee_3}+1\right)\left(\alpha\frac{1+ee_3}{1-ee_3}+\ 1\right)\left(\frac{1+ee_3}{1-ee_3}\gamma+\alpha\beta\right)}{\left(\beta+\frac{1+ee_3}{1-ee_3}\right)\left(\alpha+\ \frac{1+ee_3}{1-ee_3}\right)\left(\gamma+\alpha\beta\frac{1+ee_3}{1-ee_3}\right)} \qquad \textbf{(17)}$$

From equations **(17)** and **(10)**:

$$ee_4 = \frac{\frac{\left(\beta\frac{[3^S]}{[3^R]}+1\right)\left(\alpha\frac{[3^S]}{[3^R]}+\ 1\right)\left(\frac{[3^S]}{[3^R]}\gamma+\alpha\beta\right)}{\left(\beta+\frac{[3^S]}{[3^R]}\right)\left(\alpha+\ \frac{[3^S]}{[3^R]}\right)\left(\gamma+\alpha\beta\frac{[3^S]}{[3^R]}\right)}-1}{\frac{\left(\beta\frac{[3^S]}{[3^R]}+1\right)\left(\alpha\frac{[3^S]}{[3^R]}+\ 1\right)\left(\frac{[3^S]}{[3^R]}\gamma+\alpha\beta\right)}{\left(\beta+\frac{[3^S]}{[3^R]}\right)\left(\alpha+\ \frac{[3^S]}{[3^R]}\right)\left(\gamma+\alpha\beta\frac{[3^S]}{[3^R]}\right)}+1}$$

$$\boldsymbol{ee_4 = \frac{ee_3\alpha\beta(3+\alpha+\beta-\alpha\beta)+ee_3{}^3(\alpha-1)(\beta-1)(\alpha\beta-\gamma)-ee_3\gamma(\alpha+\beta+3\alpha\beta-1)}{\alpha\beta\left(ee_3{}^2(\alpha+\beta+\alpha\beta-3)-(\alpha+1)(\beta+1)\right)-\gamma\left(1+\alpha+\beta+\alpha\beta+ee_3{}^2(\alpha(3\beta-1)-\beta-1)\right)}} \qquad \textbf{(18)}$$

Equation **(18)** allows us to rationalize the appearance of NLE and its sense (summarized in **Figure S1**).

1. If $\alpha\beta = 1$:

$$ee_4 = \frac{ee_3\alpha\beta(3+\alpha+\beta-\alpha\beta)+ee_3{}^3(\alpha-1)(\beta-1)(\alpha\beta-\gamma)-ee_3\gamma(\alpha+\beta+3\alpha\beta-1)}{\alpha\beta\left(ee_3{}^2(\alpha+\beta+\alpha\beta-3)-(\alpha+1)(\beta+1)\right)-\gamma\left(1+\alpha+\beta+\alpha\beta+ee_3{}^2(\alpha(3\beta-1)-\beta-1)\right)} \qquad \textbf{(18)}$$

$$ee_4 = \frac{ee_3\left(3+\alpha+\frac{1}{\alpha}-1\right)+ee_3{}^3(\alpha-1)\left(\frac{1}{\alpha}-1\right)(1-\gamma)-ee_3\gamma(\alpha+\frac{1}{\alpha}+3-1)}{ee_3{}^2\left(\alpha+\frac{1}{\alpha}+1-3\right)-(\alpha+1)\left(\frac{1}{\alpha}+1\right)-\gamma\left(1+\alpha+\frac{1}{\alpha}+1+ee_3{}^2\left(\alpha\left(\frac{3}{\alpha}-1\right)-\frac{1}{\alpha}-1\right)\right)}$$

$$ee_4 = \frac{\gamma-1}{\gamma+1}ee_3 = ee_4{}^{ep}ee_3$$

In this scenario, the selectivity of the first step (α) is the inverse of the selectivity of the second step (β) and therefore they cancel each other. The result is a linear dependency of the *ee* of the product (ee_4) with respect to the *ee* of the catalyst (ee_3) (**Figure S1**).

2. If $\alpha = \gamma$:

$$ee_4 = \frac{ee_3\alpha\beta(3+\alpha+\beta-\alpha\beta) + ee_3^3(\alpha-1)(\beta-1)(\alpha\beta-\gamma) - ee_3\gamma(\alpha+\beta+3\alpha\beta-1)}{\alpha\beta\left(ee_3^2(\alpha+\beta+\alpha\beta-3) - (\alpha+1)(\beta+1)\right) - \gamma\left(1+\alpha+\beta+\alpha\beta+ee_3^2(\alpha(3\beta-1)-\beta-1)\right)} \quad \textbf{(18)}$$

$$ee_4 = \frac{ee_3\alpha\beta(3+\alpha+\beta-\alpha\beta) + ee_3^3(\alpha-1)(\beta-1)(\alpha\beta-\alpha) - ee_3\alpha(\alpha+\beta+3\alpha\beta-1)}{\alpha\beta\left(ee_3^2(\alpha+\beta+\alpha\beta-3) - (\alpha+1)(\beta+1)\right) - \alpha\left(1+\alpha+\beta+\alpha\beta+ee_3^2(\alpha(3\beta-1)-1-\beta)\right)}$$

$$ee_4 = \frac{\gamma-1}{\gamma+1}ee_3 = ee_4^{ep}ee_3$$

In this scenario, the result is a linear dependency of the ee of the product (ee_4) with respect to the ee of the catalyst (ee_3) (**Figure S1**), which may be due to different reasons. To understand the different reasons, we have used equation **(14)** and definitions **(7)** and **(8)** to derive the following equation:

$$\gamma = \frac{k_{-1}\beta + k_3\alpha}{k_{-1}+k_3} = \frac{\frac{k_{-1}}{k_3}\beta + \alpha}{\frac{k_{-1}}{k_3}+1};\ as\ \alpha = \gamma:\ \alpha = \frac{\frac{k_{-1}}{k_3}\beta + \alpha}{\frac{k_{-1}}{k_3}+1} \rightarrow \frac{k_{-1}}{k_3}\alpha = \frac{k_{-1}}{k_3}\beta$$

Which means that either the first step is practically irreversible ($k_{-1}/k_3 \approx 0$), or that both steps have identical selectivity ($\alpha = \beta$) (**Figure S1**).

3. $\beta = \gamma$:

$$ee_4 = \frac{ee_3\alpha\beta(3+\alpha+\beta-\alpha\beta) + ee_3^3(\alpha-1)(\beta-1)(\alpha\beta-\gamma) - ee_3\gamma(\alpha+\beta+3\alpha\beta-1)}{\alpha\beta\left(ee_3^2(\alpha+\beta+\alpha\beta-3) - (\alpha+1)(\beta+1)\right) - \gamma\left(1+\alpha+\beta+\alpha\beta+ee_3^2(\alpha(3\beta-1)-\beta-1)\right)} \quad \textbf{(18)}$$

$$ee_4 = \frac{ee_3\alpha\beta(3+\alpha+\beta-\alpha\beta) + ee_3^3(\alpha-1)(\beta-1)(\alpha\beta-\beta) - ee_3\beta(\alpha+\beta+3\alpha\beta-1)}{\alpha\beta\left(ee_3^2(\alpha+\beta+\alpha\beta-3) - (\alpha+1)(\beta+1)\right) - \beta\left(1+\alpha+\beta+\alpha\beta+ee_3^2(\alpha(3\beta-1)-\beta-1)\right)}$$

$$ee_4 = \frac{\gamma-1}{\gamma+1}ee_3 = ee_4^{ep}ee_3$$

In this scenario, the result is a linear dependency of the ee of the product (ee_4) with respect to the ee of the catalyst (ee_3) (**Figure S1**), which may be due to different reasons. To understand the different reasons, we have used equation **(14)** and definitions **(7)** and **(8)** to derive the following equation:

$$\gamma = \frac{k_{-1}\beta + k_3\alpha}{k_{-1}+k_3} = \frac{\beta + \frac{k_3}{k_{-1}}\alpha}{1+\frac{k_3}{k_{-1}}};\ as\ \beta = \gamma:\ \beta = \frac{\beta + \frac{k_3}{k_{-1}}\alpha}{1+\frac{k_3}{k_{-1}}} \rightarrow \frac{k_3}{k_{-1}}\alpha = \frac{k_3}{k_{-1}}\beta$$

Which means that either the first step is in quasi-equilibrium (because $k_3/k_{-1} \approx 0$) or that both steps have identical selectivity ($\alpha = \beta$) (**Figure S1**).

4. $\beta < \gamma < \alpha$:

$$ee_4 = \frac{ee_3\alpha\beta(3+\alpha+\beta-\alpha\beta) + ee_3^3(\alpha-1)(\beta-1)(\alpha\beta-\gamma) - ee_3\gamma(\alpha+\beta+3\alpha\beta-1)}{\alpha\beta\big(ee_3^2(\alpha+\beta+\alpha\beta-3)-(\alpha+1)(\beta+1)\big) - \gamma\big(1+\alpha+\beta+\alpha\beta+ee_3^2(\alpha(3\beta-1)-\beta-1)\big)} \quad \textbf{(18)}$$

As in the absense of NLE: $ee_4(linear) = \frac{\gamma-1}{\gamma+1}ee_3$, then the difference between ee_4 and $ee_4(linear)$ is:

$$ee_4 - ee_4(linear) = \frac{ee_3\alpha\beta(3+\alpha+\beta-\alpha\beta) + ee_3^3(\alpha-1)(\beta-1)(\alpha\beta-\gamma) - ee_3\gamma(\alpha+\beta+3\alpha\beta-1)}{\alpha\beta\big(ee_3^2(\alpha+\beta+\alpha\beta-3)-(\alpha+1)(\beta+1)\big) - \gamma\big(1+\alpha+\beta+\alpha\beta+ee_3^2(\alpha(3\beta-1)-\beta-1)\big)} - \frac{\gamma-1}{\gamma+1}ee_3$$

$$ee_4 - ee_4(linear) = \frac{2ee_3(1-ee_3^2)(\alpha-\gamma)(\gamma-\beta)(1-\alpha\beta)}{\delta-\varepsilon}, \text{where:} \quad \textbf{(19)}$$

$$\delta = \alpha\beta(1+\alpha+\beta+\alpha\beta) + \gamma(1+\alpha+\beta+2\alpha\beta+\alpha^2\beta+\alpha\beta^2+\alpha^2\beta^2) + \gamma^2(1+\alpha+\beta+\alpha\beta) + 3ee_3\alpha\beta(1+2\gamma+\gamma^2)$$

$$\varepsilon = ee_3^2\big(\alpha\beta(\alpha+\beta+\alpha\beta) + \gamma(1+\alpha+\beta+\alpha^2\beta+\alpha\beta^2+\alpha^2\beta^2) + \gamma^2(1+\alpha+\beta)\big)$$

As $1 \geq ee_3 \geq 0$ and the other parameters are positive: $\delta > \varepsilon$, and therefore, the denominator of **(19)** is positive.

As $1 \geq ee_3 \geq 0$, the term $2ee_3(1-ee_3^2)$ of the numerator of **(19)** is positive.

As $\beta < \gamma < \alpha$, the term $(\alpha-\gamma)(\gamma-\beta)$ of the numerator of **(19)** is positive.

Therefore, the sign of the equation **(19)** depends on the value of $(1-\alpha\beta)$:

- If $\alpha\beta > 1$, the sign of **(19)** is negative and there is (–)-NLE (**Figure S1**).
- If $\alpha\beta = 1$, **(19)** is zero and the system has a linear behaviour (see point 1 in page S-3 and **Figure S1**).
- If $\alpha\beta < 1$, the sign of **(19)** is positive and there is (+)-NLE (**Figure S1**).

5. $\beta > \gamma > \alpha$:

$$ee_4 = \frac{ee_3\alpha\beta(3+\alpha+\beta-\alpha\beta) + ee_3^3(\alpha-1)(\beta-1)(\alpha\beta-\gamma) - ee_3\gamma(\alpha+\beta+3\alpha\beta-1)}{\alpha\beta\big(ee_3^2(\alpha+\beta+\alpha\beta-3)-(\alpha+1)(\beta+1)\big) - \gamma\big(1+\alpha+\beta+\alpha\beta+ee_3^2(\alpha(3\beta-1)-1-\beta)\big)} \quad \textbf{(18)}$$

As in the absense of NLE: $ee_4(linear) = \frac{\gamma-1}{\gamma+1}ee_3$, then the difference between ee_4 and $ee_4(linear)$ is:

$$ee_4 - ee_4(linear) = \frac{ee_3\alpha\beta(3+\alpha+\beta-\alpha\beta) + ee_3^3(\alpha-1)(\beta-1)(\alpha\beta-\gamma) - ee_3\gamma(\alpha+\beta+3\alpha\beta-1)}{\alpha\beta\big(ee_3^2(\alpha+\beta+\alpha\beta-3)-(\alpha+1)(\beta+1)\big) - \gamma\big(1+\alpha+\beta+\alpha\beta+ee_3^2(\alpha(3\beta-1)-\beta-1)\big)} - \frac{\gamma-1}{\gamma+1}ee_3$$

$$ee_4 - ee_4(linear) = \frac{2ee_3(1-ee_3^2)(\alpha-\gamma)(\gamma-\beta)(1-\alpha\beta)}{\delta-\varepsilon}, \text{where:} \quad \textbf{(19)}$$

$$\delta = \alpha\beta(1+\alpha+\beta+\alpha\beta) + \gamma(1+\alpha+\beta+2\alpha\beta+\alpha^2\beta+\alpha\beta^2+\alpha^2\beta^2) + \gamma^2(1+\alpha+\beta+\alpha\beta) + 3ee_3\alpha\beta(1+2\gamma+\gamma^2)$$

$$\varepsilon = ee_3^2\big(\alpha\beta(\alpha+\beta+\alpha\beta) + \gamma(1+\alpha+\beta+\alpha^2\beta+\alpha\beta^2+\alpha^2\beta^2) + \gamma^2(1+\alpha+\beta)\big)$$

As $1 \geq ee_3 \geq 0$ and the other parameters are positive: $\delta > \varepsilon$, and therefore, the denominator of **(19)** is positive.

As $1 \geq ee_3 \geq 0$, the term $2ee_3(1-ee_3^2)$ of the numerator of **(19)** is positive.

As $\beta > \gamma > \alpha$, the term $(\alpha-\gamma)(\gamma-\beta)$ of the numerator of **(19)** is positive.

Therefore, the sign of the equation **(19)** depends on the value of $(1-\alpha\beta)$:

- If $\alpha\beta > 1$, the sign of **(19)** is negative and there is (–)-NLE (**Figure S1**).
- If $\alpha\beta = 1$, **(19)** is zero and the system has a linear behaviour (see point 1 in page S-3 and **Figure S1**).
- If $\alpha\beta < 1$, the sign of **(19)** is positive and there is (+)-NLE (**Figure S1**)

α, β, γ relationship	kinetic scenario	sense of the nonlinear effect
$\beta < \gamma < \alpha$	**lock and key**	$\alpha\beta > 1$ **(–)-nonlinear effect**<br>$\alpha\beta = 1$ **linear**<br>$\alpha\beta < 1$ **(+)-nonlinear effect**
$\alpha = \gamma \neq \beta$	**irreversible first step**	**linear**
$\beta = \gamma = \alpha$	**equal selectivity on each step**	**linear**
$\beta = \gamma \neq \alpha$	**quasi-equilibrium**	**linear**
$\beta > \gamma > \alpha$	**major-minor**	$\alpha\beta > 1$ **(–)-nonlinear effect**<br>$\alpha\beta = 1$ **linear**<br>$\alpha\beta < 1$ **(+)-nonlinear effect**

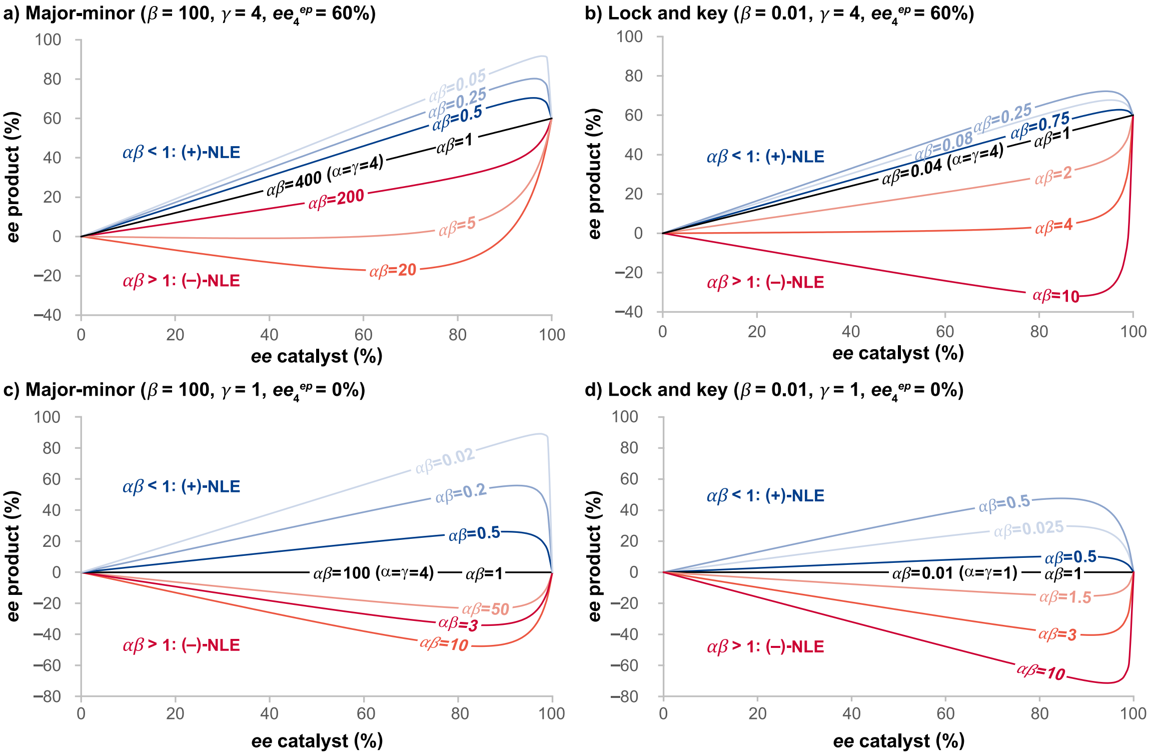

Figure S1. Diagram of the outcomes at each kinetic scenario and the resulting NLEs in each case when a) $\beta = 100$ and ee_4^{ep} = 60%. b). $\beta = 0.01$ and ee_4^{ep} = 60%. c) $\beta = 100$ and ee_4^{ep} = 0%. d) $\beta = 0.01$ and ee_4^{ep} = 0%.

3. Procedure used to obtain rate constants for the kinetic modelling of Scheme 2

To find the rate constants which produced a nonlinear effect comparable to the nonlinear effect reported in Ref. 2, we used the "solver" function in Microsoft Excel to find the values of α, β and γ. The values we found minimized the square of the differences between the value of the steady-state equation for ee_4 (formula **(18)**) and the experimental data (page S64 of Ref. 2; we considered that the result for 98% ee_3 reported by Jørgensen *et al.* was 100%, therefore the ee_4^{ep} was 0.9 and γ was 19 for all our modelling of this reaction).

$$ee_4 = \frac{ee_3\alpha\beta(3+\alpha+\beta-\alpha\beta) + ee_3{}^3(\alpha-1)(\beta-1)(\alpha\beta-\gamma) - ee_3\gamma(\alpha+\beta+3\alpha\beta-1)}{\alpha\beta\left(ee_3{}^2(\alpha+\beta+\alpha\beta-3)-(\alpha+1)(\beta+1)\right)-\gamma\left(1+\alpha+\beta+\alpha\beta+ee_3{}^2(\alpha(3\beta-1)-\beta-1)\right)} \quad \textbf{(18)}$$

For Case 1, we used the following constraint in our optimization:

$$\alpha < \gamma < \beta$$

For Case 2, we used the following constraint in our optimization:

$$\alpha > \gamma > \beta$$

We created the COPASI model representing the reaction network in Scheme 2. We assigned all the kinetic constants to global quantities defined as follows:

$$k_1 = k_1 \quad \textbf{(20)}$$

$$k_2 = \frac{k_1}{\alpha} \quad \textbf{(rearranged 7)}$$

$$k_3 = k_3 \quad \textbf{(21)}$$

$$k_4 = \frac{k_3}{\beta} \quad \textbf{(rearranged 8)}$$

$$k_{-1} = \frac{k_3k_4((1-ee_4{}^{ep})k_1 - k_2 - ee_4{}^{ep}k_2)}{k_2k_3((ee_4{}^{ep}-1)+k_4+ee_4{}^{ep}k_4)} \quad \textbf{(from 10 and 14) (22)}$$

$$k_{-2} = \frac{k_2k_{-1}}{k_1} \quad \textbf{(6)}$$

Since no numerical data nor reaction profiles were reported for the kinetic studies in Ref. 2, we had to estimate the progress of the reaction from the images of the stacked NMR spectra in pages S66-S68 in Ref 2. We used COPASI to do a "parameter estimation" of the kinetic constants that better fitted the estimated data.

4. Kinetic modelling of Scheme 2

Using the reaction scheme proposed in Scheme 2, we found two sets of rate constants which reproduced the nonlinear effect reported in Ref. 2. The first set of rate constants produce **II(*RR*)** as the major intermediate (Case 1, **Figure S2**, **Table S1**) where the reaction is under the major-minor kinetic scenario. The other set of rate constants produce **II(*SS*)** as the major intermediate (Case 2, **Figure S2**, **Table S1**) which put the reaction under the lock-and-key kinetic scenario. In both cases, the major enantiomer of product was **4(*SSSS*)**.

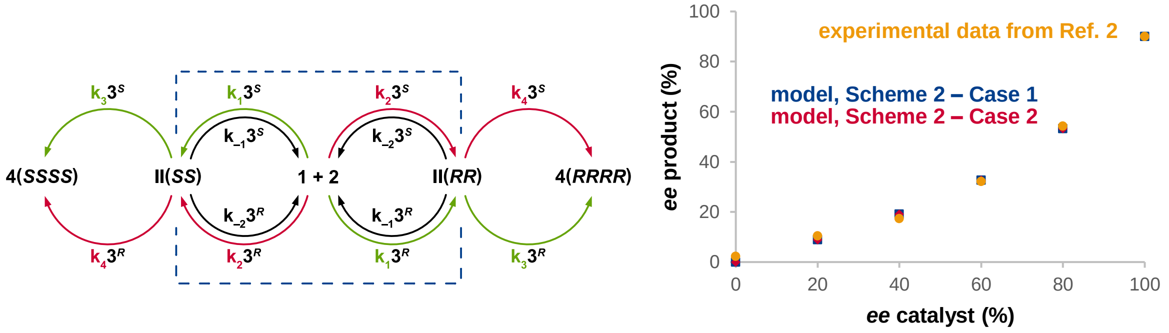

Figure S2. Nonlinear effect observed from the reaction presented in Scheme 2.

Table S1. Parameters used to model the reaction in Scheme 2 considering [**1**]$_o$ = 0.125 M, [**2**]$_o$ = 0.250 M and [**3**]$_o$ = 0.025 M.

	Case 1 (major-minor)	Case 2 (lock and key)
k_1 / M^2 min^{-1}	3.1143	9.00033
k_{-1} / min^{-1}	3.98047	9.90365
k_2 / M^2 min^{-1}	5.37969	0.158749
k_{-2} / min^{-1}	6.87591	0.174682
k_3 / M min^{-1}	8.14525	4.83977
k_4 / M min^{-1}	0.143667	8.36029
α	0.57890	56.6952
β	56.6952	0.5789
ee_4^{ep}	0.9	0.9

Provided COPASI models: "Scheme 2 Major-minor (case 1).cps" and "Scheme 2 Lock and key (case 2).cps"

The nonlinear effects shown in **Figure S2** is possible with any set of kinetic constants that maintain the values of α, β, ee_4^{ep}. To exemplify this fact, we have reproduced the same nonlinear effect with rate constants differing by a magnitude of 14 (**Figure S3**, **Table S2**).

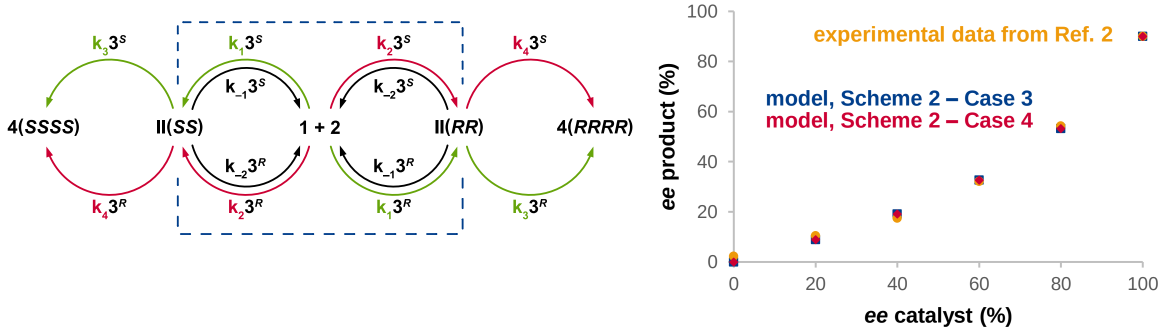

Figure S3. Nonlinear effect observed from the reaction presented in Scheme 2 using kinetic constants of Case 3 and Case 4.

Table S2. Parameters used for the modelling of **Figure S3** considering [**1**]$_o$ = 0.125 M, [**2**]$_o$ = 0.250 M and [**3**]$_o$ = 0.025 M.

	Case 3 (major-minor)	Case 4 (lock and key)
k_1 / M^2 min^{-1}	3.42	5.535
k_{-1} / min^{-1}	$7.86784 \cdot 10^{13}$	$9.20838 \cdot 10^{14}$
k_2 / M^2 min^{-1}	5.90776	$9.76273 \cdot 10^{-2}$
k_{-2} / min^{-1}	$1.3591 \cdot 10^{14}$	$1.62419 \cdot 10^{13}$
k_3 / M min^{-1}	$1.61 \cdot 10^{14}$	$4.5 \cdot 10^{14}$
k_4 / M min^{-1}	$2.83975 \cdot 10^{12}$	$7.77336 \cdot 10^{14}$
α	0.57890	56.6952
β	56.6952	0.5789
ee_4^{ep}	0.9	0.9

Provided COPASI models: "Scheme 2 Major-minor (case 3).cps" and "Scheme 2 Lock and key (case 4).cps"

To reproduce the experiment conducted by Jørgensen *et al.*, where the intermediate **II** was isolated and reacted under the same conditions as the [10+2] cycloaddition,[2] we used the parameters presented for Case 1 (**Figure S4**).

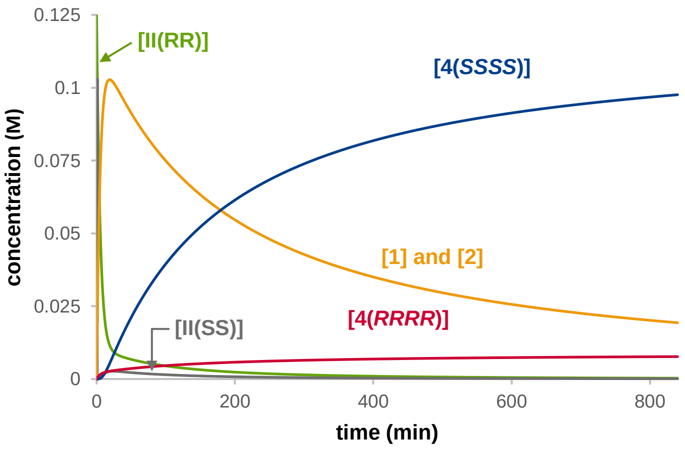

Figure S4. Formation of **1** and **4** from a reaction initiated from **II(*RR*)** in Case 1 ([**II(*RR*)**]$_o$ = 0.125 M and [**3**^{S}]$_o$ = 0.025 M).

5. Kinetic modelling of the extended reaction with unproductive diastereomeric intermediates

When the reaction in Scheme 2 is extended to include the formation of both unproductive diastereomeric intermediates (**II(*SR*)**, **II(*RS*)**), and the catalyst bound intermediates (e.g. **Int(*SS*)3*S***) we can also produce a nonlinear effect for both the kinetic scenarios in Case 1 and 2 (**Figure S5**, **Table S3)**.

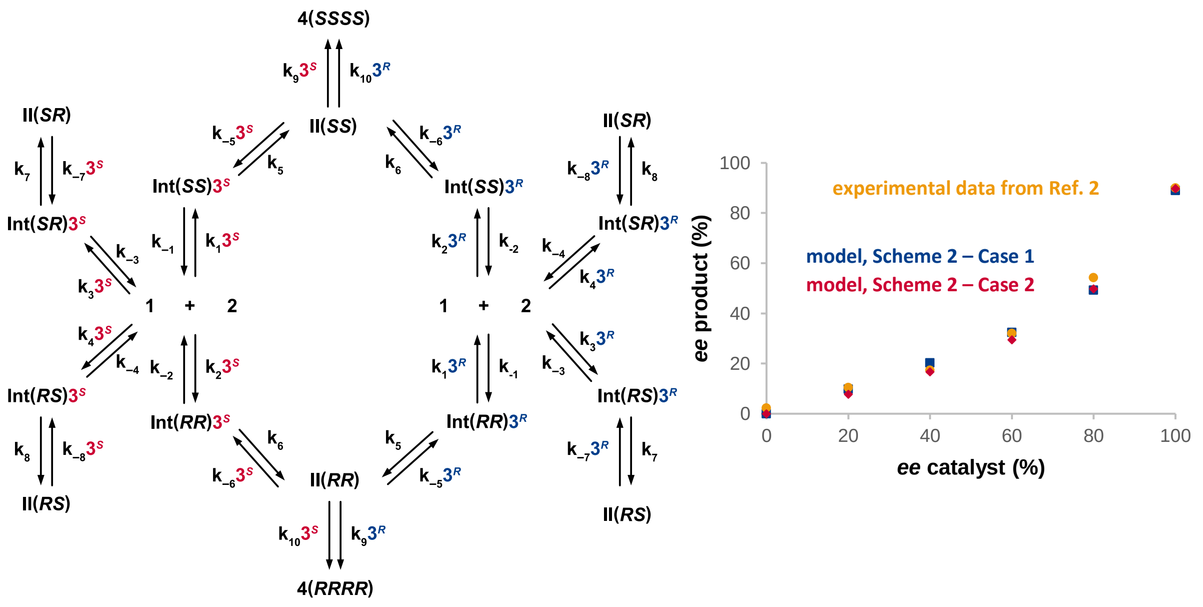

Figure S5. Nonlinear effect observed in the extended reaction.

Table S3. Parameters used for the modelling of **Figure S5** considering [**1**]o = 0.125 M, [**2**]o = 0.250 M and [**3**]o = 0.025 M.

	Case 1 (major-minor)	Case 2 (lock-and-key)
k_1 / M² min⁻¹	3.97749	8.55524
k_{-1} / min⁻¹	40.1836	$1.92127 \cdot 10^{6}$
k_2 / M² min⁻¹	20292.7	$1.14530 \cdot 10^{-1}$
k_{-2} / min⁻¹	210	$8.16313 \cdot 10^{-3}$
k_3 / M² min⁻¹	5.37749	5.85524
k_{-3} / min⁻¹	4000.18	$1.92127 \cdot 10^{6}$
k_4 / M² min⁻¹	14.5296	$1.14530 \cdot 10^{-2}$
k_{-4} / min⁻¹	$8.19613 \cdot 10^{6}$	$8.19613 \cdot 10^{-2}$
k_5 / min⁻¹	$1.82477 \cdot 10^{7}$	$2.09886 \cdot 10^{7}$
k_{-5} / M min⁻¹	$6.26530 \cdot 10^{6}$	37.3379
k_6 / min⁻¹	$2.1 \cdot 10^{-2}$	$4.87739 \cdot 10^{9}$
k_{-6} / M min⁻¹	7.03902	$2.72282 \cdot 10^{10}$
k_7 / min⁻¹	$2.09886 \cdot 10^{7}$	$1.89886 \cdot 10^{7}$
k_{-7} / M min⁻¹	100054	37.3379
k_8 / min⁻¹	2.1	$4.87739 \cdot 10^{9}$
k_{-8} / M min⁻¹	$1.32026 \cdot 10^{-5}$	$4.39741 \cdot 10^{8}$
k_9 / M min⁻¹	60.9146	1.10427
k_{10} / M min⁻¹	$7.26293 \cdot 10^{-1}$	1.32329

Provided COPASI models: “All diastereomers (case 1).cps” and “All diastereomers (case 2).cps”

6. Kinetic modelling of Scheme 2 with a completely enantiospecific catalyst in the final step

Even with a catalyst completely enantiospecific in the second step, we can produce a nonlinear effect, but only under the major-minor kinetic scenario where **II(*RR*)** is the major intermediate and **4(*SSSS*)** is the major product (**Figure S6**, **Table S4**).

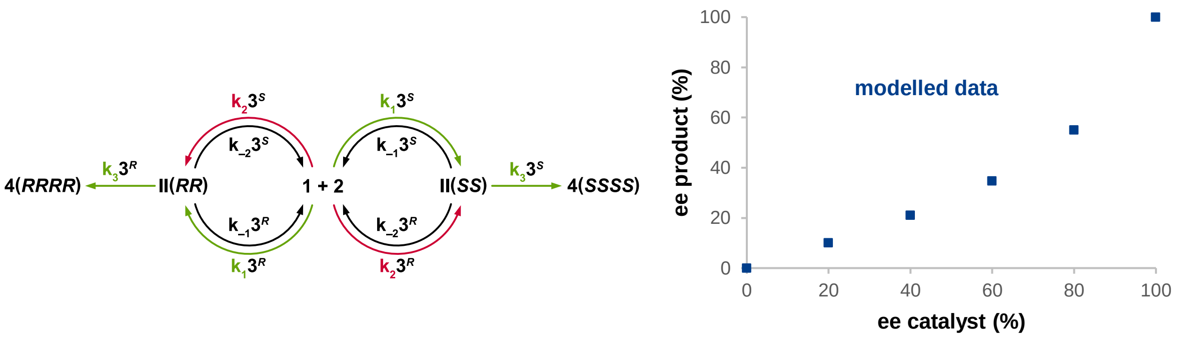

Figure S6. Nonlinear effect observed when formation of **4** from intermediate **II** is completely enantiospecific.

Table S4. Parameters used for the modelling of **Figure S6** considering [**1**]$_o$ = 0.125 M and [**2**]$_o$ = 0.250 M.

	**completely enantiospecific final step**
**k_1 / M^2 min^{-1}**	4.36707
**k_{-1} / min^{-1}**	9.08032
**k_2 / M^2 min^{-1}**	26.6285
**k_{-2} / min^{-1}**	55.3678
**k_3 / min^{-1}**	26.6285
**α**	0.1631
ee_4^{ep}	1.0

Provided COPASI models: “Scheme 2 Enantiospecific second step.cps”

7. Kinetic modelling of Scheme 2 with an uncatalyzed final step

Even with an uncatalyzed second step, we can produce a nonlinear effect, but only under the lock-and-key kinetic scenario where **II(*SS*)** is the major intermediate and **4(*SSSS*)** is the major product (**Figure S7**, **Table S5**).

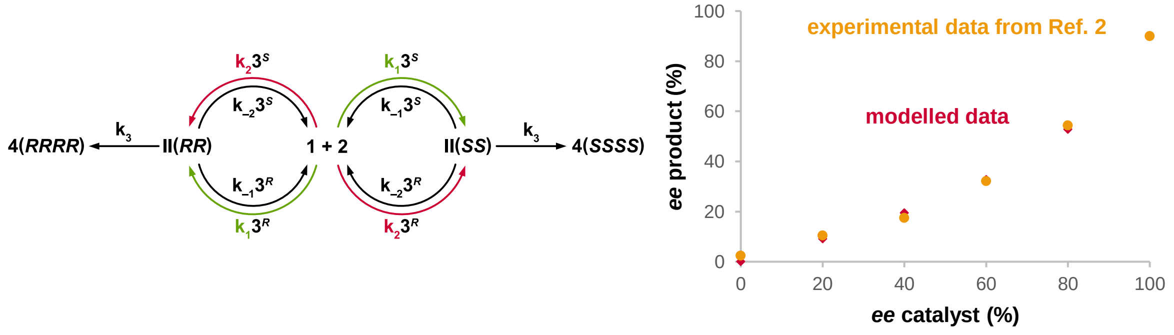

Figure S7. Nonlinear effect observed when formation of **4** from **II** is uncatalyzed.

Table S5. Parameters used for the modelling of **Figure S7** considering [**1**]ₒ = 0.125 M and [**2**]ₒ = 0.250 M.

	uncatalyzed final step
k_1 / M^2 min^{-1}	9.00123
k_{-1} / min^{-1}	18.6217
k_2 / M^2 min^{-1}	0.149642
k_{-2} / min^{-1}	0.309579
k_3 / min^{-1}	0.203631
α	60.1517
β	1
ee_4^{ep}	0.9

Provided COPASI models: "Scheme 2 Uncatalyzed second step.cps"

8. Measurement of the order in catalyst for Scheme 2

To measure the order in catalyst for the reaction network shown in Scheme 2 we used time normalised analysis[3] on reaction profiles generated in COPASI. We used the reaction conditions used in the experimental kinetic studies reported in the supporting information of Ref. 2 (page S65). Using these conditions, we measured an order of 1 in catalyst (**Figure S8**).

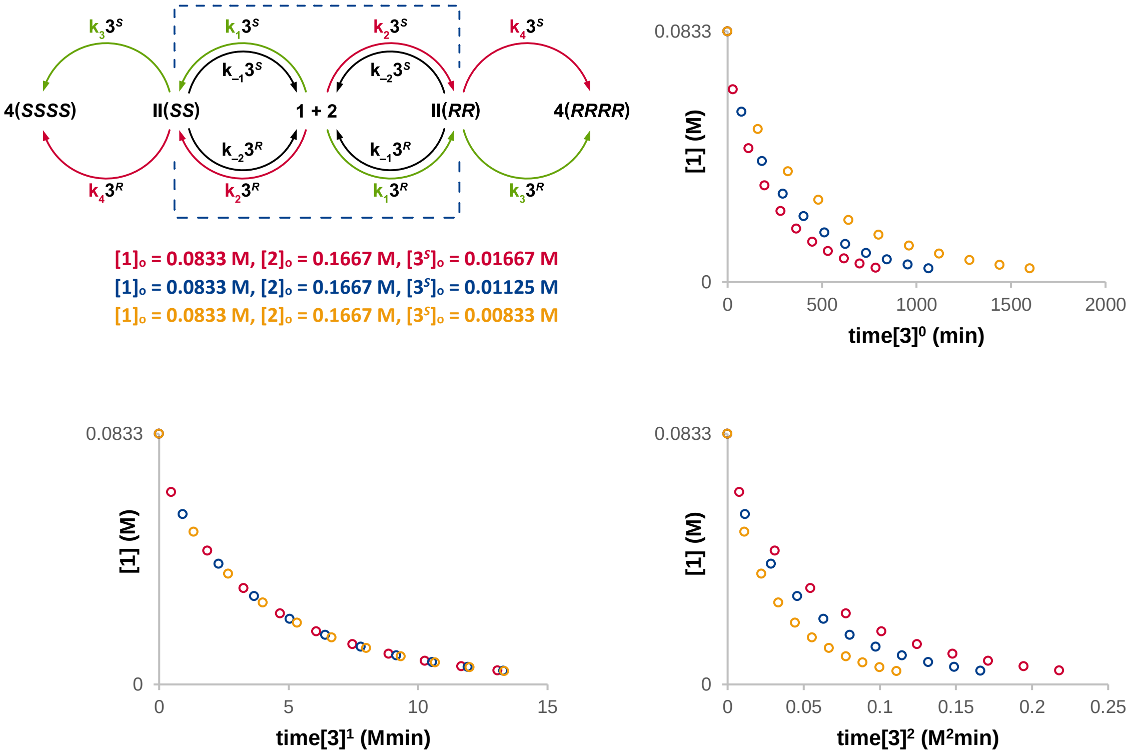

Figure S8. Order in catalyst is 1 for the reaction presented in Scheme 2.

We also derived the order in catalyst expected from the reaction pathway shown in Scheme 2:

Rate of formation of **4**:

$$r_{\mathbf{4}} = (k_3[3^S]+k_4[3^R])[\mathrm{II}(SS)] + (k_3[3^R]+k_4[3^S])[\mathrm{II}(RR)] \qquad \textbf{(2)}$$

Steady-state approximation for **II(*SS*)**:

$$(k_1[3^S] + k_2[3^R])[1][2] - (k_{-1}[3^S] + k_{-2}[3^R] + k_3[3^S]+k_4[3^R])[\mathrm{II}(SS)] \approx 0 \qquad \textbf{(4)}$$

Steady-state approximation for **II(*RR*)**:

$$(k_1[3^R] + k_2[3^S])[1][2] - (k_{-1}[3^R] + k_{-2}[3^S] + k_3[3^R]+k_4[3^S])[\mathrm{II}(RR)] \approx 0 \qquad \textbf{(5)}$$

From equations **(2)**, **(4)**, and **(5)** we derive the rate of formation of **4** in terms of **1** and **2**:

$$r_4 = [1][2](\frac{(k_1[3^S] + k_2[3^R])(k_3[3^S] + k_4[3^R])}{[3^S](k_{-1} + k_3) + [3^R](k_{-2} + k_4)} + \frac{(k_1[3^R] + k_2[3^S])(k_3[3^R] + k_4[3^S])}{[3^R](k_{-1} + k_3) + [3^S](k_{-2} + k_4)}) \qquad \textbf{(23)}$$

Mass Balance of **3:**

$$[3] = [3^S] + [3^R] \qquad \textbf{(24)}$$

Enantiomeric ratio of catalyst **3**:

$$er_3 = \frac{[3^S]}{[3^R]} \qquad \textbf{(9)}$$

From equations **(9)**, **(24)**, and **(23):**

$$r_{\mathbf{4}} = \mathrm{a}[3];\ \text{where a} =$$

$$[1][2]\,\frac{(k_1 + k_2)(er_3k_3 + k_4)(k_3 + k_4er_3) + k_1(er_3k_3(k_1 + k_2) + (er_3k_1 + (1 + (er_3 - 1)er_3)k_2)k_4) + k_{-2}\big(er_3k_2(k_3 + k_4) + k_1\big(k_4(1 + er_3{}^2 - er_3)\big) + er_3k_4\big)}{(k_{-2} + er_3(k_{-1} + k_3) + k_4)\big(k_{-1} + k_3 + er_3(k_{-2} + k_4)\big)} \qquad \textbf{(25)}$$

9. Procedure for the modelling of Figure 5

For both Case 1 and 2, we plotted the ratio of intermediates [**II(RR)**]/[**II(SS)**] at the end of each simulated reaction profiles ("**Scheme 2 Major-minor (case 1)**" and "**Scheme 2 Lock and key (case 2)**") against the *ee* of the catalyst (*ee3*).

10. Kinetic analysis of the mechanism shown in Ref. 2.

We represented the mechanism proposed in Ref. 2 (Figure 5) in **Scheme S3**. For our kinetic analysis of **Scheme S3**, we derived the equation for the *ee* of the product (*ee4*) considering the possible formation of all the catalytic species containing two units of the same enantiomer of the catalyst. Following the description of the system in Ref. 2, we have imposed the equilibrium between all the intermediates referred as **IV** in Ref. 2). We represented the stereoisomeric intermediates $\mathbf{IV}_{RS}$, $\mathbf{IV}_{SR}$ and $\mathbf{IV}_{RR}$ (from Ref. 2) as ***SS*–3*^S*3*^S***, ***RR*–3*^S*3*^S*** and ***SR*–3*^S*3*^S***.

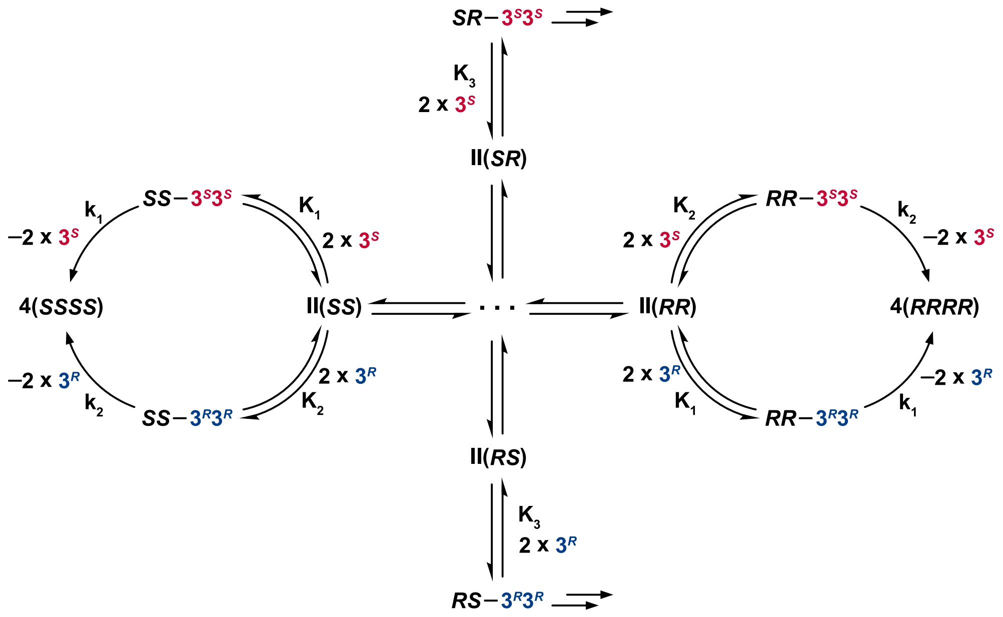

Scheme S3. Reaction network used for the mathematical analysis of the mechanism shown in Ref. 2 (Figure 5).

We used the following definitions and considered that **IV** is fully equilibrated in our derivation of the *ee* of the product (*ee4*):

Enantiomeric excess of product **4**:

$$ee_4 = \frac{[4(SSSS)] - [4(RRRR)]}{[4(SSSS)] + [4(RRRR)]} \quad \textbf{(10)}$$

Rate of formation of **4(*SSSS*)**:

$$r_{4(SSSS)} = k_1[SS - 3^S3^S] + k_2[SS - 3^R3^R] \quad \textbf{(26)}$$

Rate of formation of **4(*RRRR*)**:

$$r_{4(RRRR)} = k_1[RR - 3^R3^R] + k_2[RR - 3^S3^S] \quad \textbf{(27)}$$

Equilibrium of formation of ***SS* – 3*^S*3*^S***:

$$K_1 = \frac{[SS - 3^S3^S]_{eq}}{[\mathrm{II}(SS)]_{eq}[3^S]^2} \quad \textbf{(28)}$$

Equilibrium of formation of ***SS* – 3*^R*3*^R***:

$$K_2 = \frac{[SS - 3^R3^R]_{eq}}{[\mathrm{II}(SS)]_{eq}[3^R]^2} \quad \textbf{(29)}$$

Equilibrium of formation of ***RR* – 3*^R*3*^R***:

$$K_1 = \frac{[RR - 3^R3^R]_{eq}}{[\mathrm{II}(RR)]_{eq}[3^R]^2} \quad \textbf{(30)}$$

Equilibrium of formation of ***RR* – 3^{S}3^{S}**:

$$K_2 = \frac{[RR - 3^S3^S]_{eq}}{[\mathrm{II}(RR)]_{eq}[3^S]^2} \quad \textbf{(31)}$$

Equilibrium of formation of catalytic intermediates leading to diastereomeric products ***SR* – 3^{S}3^{S}**:

$$K_3 = \frac{[SR - 3^S3^S]_{eq}}{[\mathrm{II}(SR)]_{eq}[3^S]^2} \quad \textbf{(32)}$$

Equilibrium of formation of catalytic intermediates leading to diastereomeric products ***RS* – 3^{R}3^{R}**:

$$K_3 = \frac{[RS - 3^R3^R]_{eq}}{[\mathrm{II}(RS)]_{eq}[3^R]^2} \quad \textbf{(33)}$$

Mass balance for the catalyst **3**:

$$[3]_T = [3^S]_T + [3^R]_T \quad \textbf{(34)}$$

Mass balance for the catalyst **3^{S}**:

$$[3^S]_T = [3^S] + 2\,[SS - 3^S3^S]_{eq} + 2\,[RR - 3^S3^S]_{eq} + 2\,[SR - 3^S3^S]_{eq} \quad \textbf{(35)}$$

Mass balance for the catalyst **3^{R}**:

$$[3^R]_T = [3^R] + 2\,[SS - 3^R3^R]_{eq} + 2\,[RR - 3^R3^R]_{eq} + 2\,[RS - 3^R3^R]_{eq} \quad \textbf{(36)}$$

Enantiomeric excess of catalyst **3**:

$$ee_3 = \frac{[3^S]_T - [3^R]_T}{[3^S]_T + [3^R]_T} \quad \textbf{(37)}$$

$\mathrm{II}(SS)$ and $\mathrm{II}(RR)$ are enatiomers, therefore: $[\mathrm{II}(SS)]_{eq} = [\mathrm{II}(RR)]_{eq}$ **(38)**

$\mathrm{II}(SR)$ and $\mathrm{II}(RS)$ are enatiomers, therefore: $[\mathrm{II}(RS)]_{eq} = [\mathrm{II}(SR)]_{eq}$ **(39)**

From equations **(10)** and **(26-31)**:

$$ee_4 = \frac{k_1[SS - 3^S3^S] + k_2[SS - 3^R3^R] - k_1[RR - 3^R3^R] - k_2[RR - 3^S3^S]}{k_1[SS - 3^S3^S] + k_2[SS - 3^R3^R] + k_1[RR - 3^R3^R] + k_2[RR - 3^S3^S]}$$

$$= \frac{k_1K_1[\mathrm{II}(SS)]_{eq}[3^S]^2 + k_2K_2[\mathrm{II}(SS)]_{eq}[3^R]^2 - k_1K_1[\mathrm{II}(RR)]_{eq}[3^R]^2 - k_2K_2[\mathrm{II}(RR)]_{eq}[3^S]^2}{k_1K_1[\mathrm{II}(SS)]_{eq}[3^S]^2 + k_2K_2[\mathrm{II}(SS)]_{eq}[3^R]^2 + k_1K_1[\mathrm{II}(RR)]_{eq}[3^R]^2 + k_2K_2[\mathrm{II}(RR)]_{eq}[3^S]^2} \quad \textbf{(40)}$$

From equations **(38)** and **(40)**:

$$ee_4 = \frac{k_1K_1[3^S]^2 + k_2K_2[3^R]^2 - k_1K_1[3^R]^2 - k_2K_2[3^S]^2}{k_1K_1[3^S]^2 + k_2K_2[3^R]^2 + k_1K_1[3^R]^2 + k_2K_2[3^S]^2} = \frac{k_1K_1 - k_2K_2}{k_1K_1 + k_2K_2}\,\frac{[3^S]^2 - [3^R]^2}{[3^S]^2 + [3^R]^2} \quad \textbf{(41)}$$

From equation **(41)** we derive the *ee4* when using enantiomerically pure catalyst **3^{S}**:

$$ee_4{}^{ep} = \frac{k_1K_1 - k_2K_2}{k_1K_1 + k_2K_2}\,\frac{[3]_T{}^2}{[3]_T{}^2} = \frac{k_1K_1 - k_2K_2}{k_1K_1 + k_2K_2} \quad \textbf{(42)}$$

From equations **(41)** and **(42)**, we can write *ee4* in terms of *ee4^{ep}*, [**3^{S}**], and [**3^{R}**]:

$$ee_4 = ee_4{}^{ep}\,\frac{[3^S]^2 - [3^R]^2}{[3^S]^2 + [3^R]^2} \quad \textbf{(43)}$$

From equations **(28)**, **(31)**, **(32)** and **(35)**:

$$\left(2\,K_1[\mathrm{II}(SS)]_{eq} + 2\,K_2[\mathrm{II}(RR)]_{eq} + 2\,K_3[\mathrm{II}(SR)]_{eq}\right)[3^S]^2 + [3^S] - [3^S]_T = 0$$

$$[3^S] = \frac{2\,[3^S]_T}{1 + \sqrt{1 + 4\left(2\,K_1[\mathrm{II}(SS)]_{eq} + 2\,K_2[\mathrm{II}(RR)]_{eq} + 2\,K_3[\mathrm{II}(SR)]_{eq}\right)[3^S]_T}} \quad \textbf{(44)}$$

From equations **(29)**, **(30)**, **(33)** and **(36)**:

$$\left(2\,K_2[\mathrm{II}(SS)]_{eq} + 2\,K_1[\mathrm{II}(RR)]_{eq} + 2\,K_3[\mathrm{II}(RS)]_{eq}\right)[3^R]^2 + [3^R] - [3^R]_T = 0$$

$$[3^R] = \frac{2\,[3^R]_T}{1 + \sqrt{1 + 4\left(2\,K_2[\mathrm{II}(SS)]_{eq} + 2\,K_1[\mathrm{II}(RR)]_{eq} + 2\,K_3[\mathrm{II}(RS)]_{eq}\right)[3^R]_T}} \quad \textbf{(45)}$$

From equations **(34)** and **(37)**:

$$[3^S]_T = \frac{(1 + ee_3)}{2}[3]_T \quad \textbf{(46)}$$

$$[3^R]_T = \frac{(1 - ee_3)}{2}[3]_T \quad \textbf{(47)}$$

From equations **(38)**, **(44)** and **(46)**:

$$[3^S] = \frac{(1 + ee_3)[3]_T}{1 + \sqrt{1 + 2\,a\,[3]_T\,(1 + ee_3)}}\ \textit{where}\ a = 2\,(K_1 + K_2)[\mathrm{II}(SS)]_{eq} + 2\,K_3[\mathrm{II}(SR)]_{eq} \quad \textbf{(48)}$$

From equations **(38)**, **(39)**, **(45)** and **(47)**:

$$[3^R] = \frac{(1 - ee_3)[3]_T}{1 + \sqrt{1 + 2\,a\,[3]_T\,(1 - ee_3)}}\ \textit{where}\ a = 2\,(K_1 + K_2)[\mathrm{II}(SS)]_{eq} + 2\,K_3[\mathrm{II}(SR)]_{eq} \quad \textbf{(49)}$$

From equations **(43)**, **(48)** and **(49)**:

$$\boldsymbol{ee_4 = \frac{(1 + ee_3)^2\left(1 + \sqrt{1 + 2\,a\,[3]_T\,(1 - ee_3)}\right)^2 - (1 - ee_3)^2\left(1 + \sqrt{1 + 2\,a\,[3]_T\,(1 + ee_3)}\right)^2}{(1 + ee_3)^2\left(1 + \sqrt{1 + 2\,a\,[3]_T\,(1 - ee_3)}\right)^2 + (1 - ee_3)^2\left(1 + \sqrt{1 + 2\,a\,[3]_T\,(1 + ee_3)}\right)^2}\,{ee_4}^{ep}} \quad \textbf{(50)}$$

$$\textit{where}\ a = 2\,(K_1 + K_2)[\mathrm{II}(SS)]_{eq} + 2\,K_3[\mathrm{II}(SR)]_{eq}$$

From equations **(48)** and **(49)** the fraction of catalyst as a monomer is:

$$f_{mono} = \frac{[3^S] + [3^R]}{[3]_T} = \frac{\sqrt{1 + 2\,a\,[3]_T\,(1 + ee_3)} + \sqrt{1 + 2\,a\,[3]_T\,(1 - ee_3)} - 2}{2\,a\,[3]_T} \quad \textbf{(51)}$$

$$\textit{where}\ a = 2\,(K_1 + K_2)[\mathrm{II}(SS)]_{eq} + 2\,K_3[\mathrm{II}(SR)]_{eq}$$

From equations **(50)** and **(51)**, in the limiting case when *b* trends to zero:

$$b = 2\left(2\,(K_1 + K_2)[\mathrm{II}(SS)]_{eq} + 2\,K_3[\mathrm{II}(SR)]_{eq}\right)[3]_T$$

$$\lim_{b\to 0} ee_4 = \lim_{b\to 0}\left(\frac{(1 + ee_3)^2\left(1 + \sqrt{1 + b\,(1 - ee_3)}\right)^2 - (1 - ee_3)^2\left(1 + \sqrt{1 + b\,(1 + ee_3)}\right)^2}{(1 + ee_3)^2\left(1 + \sqrt{1 + b\,(1 - ee_3)}\right)^2 + (1 - ee_3)^2\left(1 + \sqrt{1 + b\,(1 + ee_3)}\right)^2}\,{ee_4}^{ep}\right) = \frac{2\,ee_3}{1 + {ee_3}^2}\,{ee_4}^{ep}$$

$$\lim_{b\to 0} f_{mono} = \lim_{b\to 0}\left(\frac{\sqrt{1 + b\,(1 + ee_3)} + \sqrt{1 + b\,(1 - ee_3)} - 2}{b}\right) = 1$$

From equations **(50)** and **(51)**, in the limiting case when *b* trends to infinity:

$$b = 2\left(2\,(K_1 + K_2)[\mathrm{II}(SS)]_{eq} + 2\,K_3[\mathrm{II}(SR)]_{eq}\right)[3]_T$$

$$\lim_{b\to \infty} ee_4 = \lim_{b\to \infty}\left(\frac{(1 + ee_3)^2\left(1 + \sqrt{1 + b\,(1 - ee_3)}\right)^2 - (1 - ee_3)^2\left(1 + \sqrt{1 + b\,(1 + ee_3)}\right)^2}{(1 + ee_3)^2\left(1 + \sqrt{1 + b\,(1 - ee_3)}\right)^2 + (1 - ee_3)^2\left(1 + \sqrt{1 + b\,(1 + ee_3)}\right)^2}\,{ee_4}^{ep}\right) = ee_3\,{ee_4}^{ep}$$

$$\lim_{b\to \infty} f_{mono} = \lim_{b\to \infty}\left(\frac{\sqrt{1 + b\,(1 + ee_3)} + \sqrt{1 + b\,(1 - ee_3)} - 2}{b}\right) = 0$$

11. References

1. Hoops, S.; Sahle, S.; Gauges, R.; Lee, C.; Pahle, J; Simus, N.; Singhal, M.; Xu, L.; Mendes, P.; Kummer, U. COPASI: a COmplex PAthway SImulator. *Bioinformatics* **2006**, *22* (24), 3067–3074.
2. McLeod, D.; Izzo, J. A.; Jørgensen, D. K. B.; Lauridsen, R. F.; Jørgensen, K. A. Development and Investigation of an Organocatalytic Enantioselective [10+2] Cycloaddition. *ACS Catal.* **2020**, *10*, 10784–10793.
3. Nielsen, C. D, -T.; Burés, J. Visual kinetic analysis. *Chem. Sci.* **2019**, *10*, 348–353.
